# PDZ and LIM Domain-Encoding Genes: Molecular Interactions and their Role in Development

**DOI:** 10.1100/tsw.2007.232

**Published:** 2007-09-01

**Authors:** Aartjan J. W. te Velthuis, Christoph P. Bagowski

**Affiliations:** ^1^ Department of Molecular and Cellular Biology, Institute of Biology, Leiden University, 2333 AL Leiden, The Netherlands; ^2^ Department of Integrative Zoology, Institute of Biology, Leiden University, 2333 AL Leiden, The Netherlands; ^3^ Sir William Dunn School of Pathology, University of Oxford, South Parks Road, Oxford OX1 3RE, UK

**Keywords:** PDZ, LIM domain, Enigma, LMP1, PDLIM7, ENH, Enigma homolog, PDLIM5, ZASP, Cypher, LDB3, LIM domain kinase, LIMK1, LIMK2, RIL, PDLIM4, Elfin, CLP36, PDLIM1, Mystique, PDLIM2, SLIM, ALP, PDLIM3, LMO7, PCD1, FBXO20, actin cytoskeleton, E3 ligase, NF-ĸB

## Abstract

PDZ/LIM genes encode a group of proteins that play very important, but diverse, biological roles. They have been implicated in numerous vital processes, e.g., cytoskeleton organization, neuronal signaling, cell lineage specification, organ development, and oncogenesis.

In mammals, there are ten genes that encode for both a PDZ domain, and one or several LIM domains: four genes of the ALP subfamily (ALP, Elfin, Mystique, and RIL), three of the Enigma subfamily (Enigma, Enigma Homolog, and ZASP), the two LIM kinases (LIMK1 and LIMK2), and the LIM only protein 7 (LMO7). Functionally, all PDZ and LIM domain proteins share an important trait, i.e., they can associate with and/or influence the actin cytoskeleton.

We review here the PDZ and LIM domain—encoding genes and their different gene structures, their binding partners, and their role in development and disease. Emphasis is laid on the important questions: why the combination of a PDZ domain with one or more LIM domains is found in such a diverse group of proteins, and what role the PDZ/LIM module could have in signaling complex assembly and localization.

Furthermore, the current knowledge on splice form specific expression and the function of these alternative transcripts during vertebrate development will be discussed, since another source of complexity for the PDZ and LIM domain—encoding proteins is introduced by alternative splicing, which often creates different domain combinations.

### INTRODUCTION

All fundamental biological processes largely depend on proper cellular signaling. Inaccuracies in signaling processes can have detrimental consequences, and can result in severe developmental defects and a vast variety of other pathologies.

One of the key requisites that is important for accurate signal transduction is the correct assembly of signaling complexes[1]. The temporal and spatial assembly of these multiprotein complexes (often referred to as “signalosomes”) is regulated by the interplay of different interaction domains. Especially important here are scaffold proteins, which often contain more than one interaction domain. Examples for these interaction domains are the PDZ domain (which is the most abundant protein interaction domain encoded in the human genome), and the slightly smaller and less abundant LIM domain.

Originally, PDZ domains were recognized in the postsynaptic density protein PSD-95[2], the septate junction protein Discs-large of *Drosophila melanogaster*[3], and the epithelial tight junction protein ZO-1[4]. PDZ domains play important roles in organizing cell signaling assemblies[1] and are found in plants, yeast, bacteria, and a variety of metazoans[5,6]. They recognize short C-terminal peptide motifs, internal sequences resembling a C-terminus, and have further been shown to bind to phospholipids[1,7].

The LIM domain is defined by a cysteine-rich consensus, which forms the basis for two closely associated zinc fingers. Although closely resembling DNA binding domains, nucleotide binding has never been observed and, therefore, LIM domains are now regarded as protein-interaction modules only[8,9,10]. However, in contrast to the PDZ domain, distinctively defined recognition motifs for the LIM domains have so far not been described. After its initial identification in three developmentally regulated homeodomain proteins, several studies have since revealed that LIM domain–containing proteins are implicated in a large variety of different functions, e.g., cytoskeleton organization, cell lineage specification, organ development, and oncogenesis[8,9,10].

Often, protein interaction domains like the PDZ and the LIM domains are found in proteins in combination with other functional domains or motifs. PDZ as well as LIM domain proteins often enclose multiple copies of these interaction domains. A combination of PDZ and LIM domains can also be found and, altogether, ten genes have been discovered in the mammalian genome that share the characteristic of containing both a PDZ and one or several LIM domains. These ten genes are: actinin-associated LIM protein (ALP, PDLIM3), Elfin (CLP36, PDLIM1), Enigma (LMP-1, PDLIM7), Enigma homologue (ENH, PDLIM5), reversion-induced LIM protein (RIL, PDLIM4), Mystique (PDLIM2, SLIM), Z-band associated protein (ZASP, Cypher, Oracle, PDLIM6), the two LIM domain kinases (LIMK1, LIMK2), and LIM-domain only 7 (LMO7, FBXO20) that, despite its name, also contains a PDZ domain.

### EMERGENCE OF THE PDZ AND LIM DOMAIN–ENCODING GENES

The first gene encoding for both domains was discovered in 1994 by Wu and Gill[11]. They named it *Enigma*, in reference to the deciphering of codes generated by the Enigma machines used during World War II[11]. That same year, the first of the two LIM kinase genes was discovered, encoding a protein with two LIM domains, one PDZ and a single kinase domain[12]. A year later, *RIL,* the first member containing one PDZ and a single LIM domain, was identified[13].

Some of the PDZ/LIM genes encode for additional motifs and functional domains, such as the ZASP-like motif (ZM), a conserved protein motif first identified in ZASP that has been found involved in interactions with α-actinin[14,15]; the ALP-like (AM) motif, which has been found as a conserved motif in ALP, Elfin, Mystique, and RIL[16]; a kinase domain in LIMK1 and 2; and a Calponin homology (CH) domain in LMO7 that canonically can bind to actin[17].

The PDZ/LIM genes can be divided into four different subgroups based on their gene structures and phylogenetic relationships: the ALP subfamily (ALP, RIL, Elfin, and Mystique), the Enigma subfamily (Enigma, ENH, and ZASP), the LIM kinases (LIMK1 and LIMK2), and LMO7[16]. An overview of the groups and the different human gene architectures is shown in Figs. 1–4. Recently, we investigated the evolution of the PDZ and LIM domain–encoding genes through phylogenetic analyses of sequences obtained from many different invertebrate and vertebrate species. Important insights that resulted from this research were the close evolutionary link between the Enigma (a single PDZ and three LIM domains) and ALP (one PDZ and a single LIM domain) subfamilies via a gene encoding for one N-terminal PDZ domain and four LIM domains, and the possibility of an evolutionary link of all PDZ/LIM family members via a gene encoding a single N-terminal PDZ and C-terminal LIM domain. From this ancestral gene, the LIMKs could have split off and lost their PDZ domain, only to regain it later in evolution at the other terminus. Indeed, based on our analysis of eukaryote genomes, we hypothesized that the LIMK structure was the latest to evolve within the PDZ/LIM gene family, since we were not able to identify the gene in species more basal than the fruit fly[16].

All PDZ and LIM domain–encoding proteins have been shown to be able to associate with and/or influence the actin cytoskeleton[15,18,19,20,21]. Most interactions of PDZ/LIM proteins with the cytoskeleton have been identified in skeletal muscle tissue, where several PDZ/LIM proteins are predominantly expressed[14,22,23,24]. The ALP and Enigma subfamily gene products are, together with LMO7, able to bind α-actinin via their PDZ domains[23,25,26]. The LIM kinases are more widely expressed, however, and are known for their critical role in regulating actin polymerization, functioning downstream of the Rho GTPase cascade, and influencing the activity of the cofilin family[21,27,28,29,30]. In this review, we will discuss all ten genes by subgroups and focus on their gene structures, gene expression, their binding partners, and their role in development and disease.

### THE ENIGMA SUBFAMILY (ENIGMA, ENH, AND ZASP)

The three mammalian proteins of the Enigma subfamily — Enigma itself, ENH, and ZASP — encode a single amino-terminal PDZ domain followed by three carboxy-terminal LIM domains. Within the subfamily, the *Enigma* gene was the first to be identified in mammals[11], followed by ENH[31] and ZASP[22,32]. Different roles of the three Enigma subfamily members in vertebrate development have been described. A role in heart as well as in muscle development in vertebrates has been well documented for ZASP, and mutations in ZASP have been associated with cardiac and muscular myopathies in humans[33,34,35]. Enigma and ENH, on the other hand, have not been linked to cardiac nor skeletal muscle development so far.

Enigma has been shown to be important for bone morphogenesis[36], while it has been suggested that ENH could be involved in schizophrenia[37].

#### Genomic Structure and Alternative Splicing

In the human genome, the *ENIGMA* gene is found on chromosome 5 (5q35.3) and spans 24 kbp, in total coding for 15 exons[38]. ENH covers 18 exons at position 4q22, while ZASP has been attributed the gene locus 10q22.2-q23.3 and covers approximately 70 kbp[22,39] (see Fig. 1A for the different gene architectures). For many proteins, including PDZ domain–containing proteins, alternative splicing is an important mechanism to generate functional diversity[40]. The fact that PDZ/LIM genes encode for different domains allows for alternative processing that can generate different combinations of these domains and motifs. A good example is the *ZASP* gene, which encodes for a PDZ, a LIM domain, and a ZM motif. In human heart and skeletal muscle, at least three different alternatively spliced forms of *ZASP* have been identified. A fourth form is observed in the brain, while other forms have been found in fetal

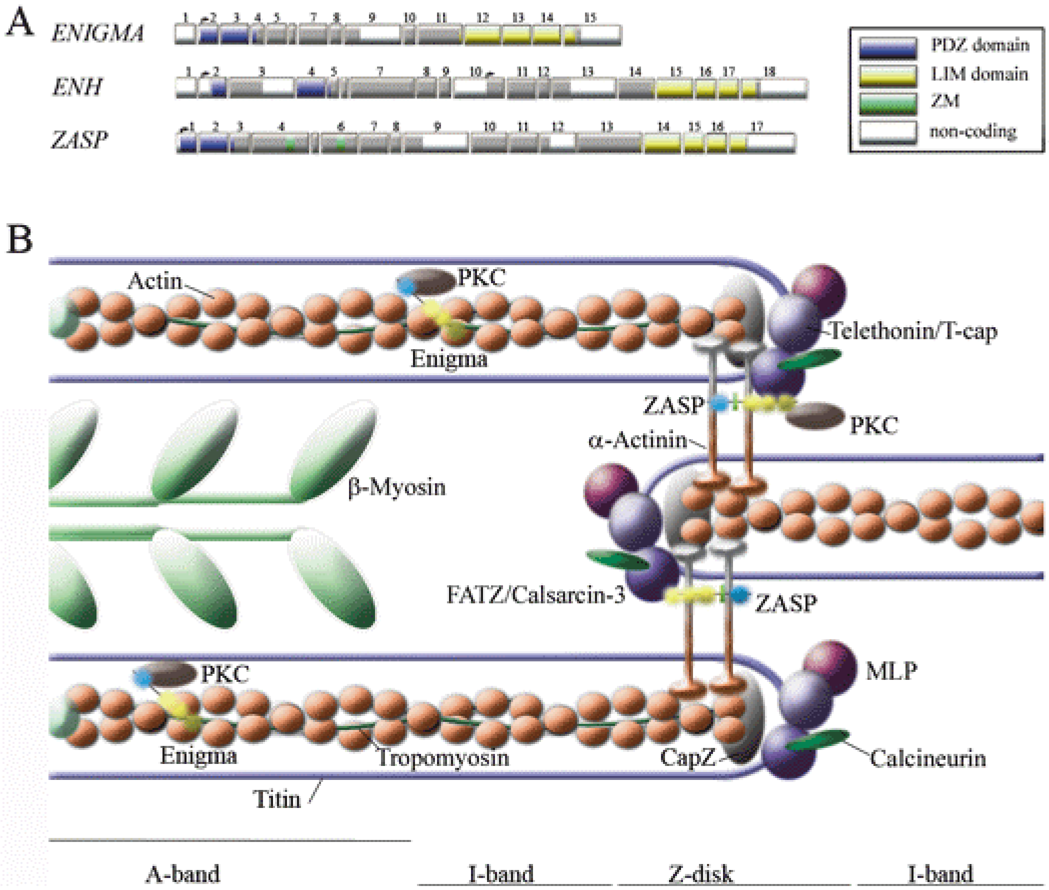

**FIGURE 1**. (A) The gene structures of the Enigma subfamily. Shown are the gene structures of the human Enigma subfamily genes based on Ensembl data. Illustrated are *ZASP* (ENSG00000122367), *ENIGMA* (ENSG00000196923), *ENH* (ENSG00000163110). In blue and yellow, the exons encoding the PDZ and LIM domain are indicated, respectively. In green, the ZASP-like motif is pointed out. (B) The Enigma subfamily and its interaction with the cytoskeleton. A simplified overview of the proteins present at the Z-disk and the I-band of the sarcomere. The major sarcomere protein titin interacts with telethonin at a 2:1 ratio and is further in complex with α-actinin[153]. The teletonin-titin complex attracts proteins like MLP and calsarcin-3 (FATZ). The actin filaments from two antiparallel sarcomeres are interconnected by α-actinin, while further cross-linking is mediated by ZASP, which interacts with α-actinin, PKC, and calsarcin-3. PKC tethering to the sarcomere is important for functional regulation, such as force generation and mechanical integrity. Enigma also interacts with PKC, but is located at a different position due to the targeting of its PDZ domain to tropomyosin. Phosphorylation of troponin (present in close association with the actin filaments and tropomyosin; not shown here for simplicity) has been shown to be essential for $Ca^{2+}$ sensitivity of muscle contraction[49,50].

lung tissue and in the pancreas[22]. Six alternatively spliced forms of murine *ZASP* have been described to date, with expression predominantly observed in cardiac muscle and skeletal muscle, and a weak expression in the lung[23]. In zebrafish, 13 variants of *ZASP* were found[34].

For ENH, four splice forms have been described including a larger variant, ENH1, containing a NH2-terminal PDZ domain and three COOH-terminal LIM motifs. ENH1 was found to be expressed in various tissues, such as heart, brain, spleen, liver, and kidney, whereas a shorter splice form was found specifically expressed in cardiac and skeletal muscles[20,41,42]. Two additional ENH splice forms, ENH2 and ENH3, lacking the three LIM motifs, have been also described in mouse[20]. It was an interesting observation that these splice forms were more widely and abundantly expressed (ratio 1:5) in several cell types. Additionally, the ratio between the largest ENH-encoding transcript and the shorter transcripts appeared to fluctuate over several embryonic stages[20]. Nakagawa et al. speculated that these ENH forms might play a dominant-negative role toward ENH1, possibly through out-competing the PDZ docking sites (i.e., α-actinin), ultimately resulting in inhibition of scaffold formation between the PDZ and LIM binding proteins (e.g., PKC)[20]. However, to the best of our knowledge currently no experimental evidence is supporting this hypothesis.

### Molecular Interactions

The currently described molecular interactions of the Enigma protein are diverse. Yeast two-hybrid screens have shown that the second LIM domain of Enigma interacts with RET/PTC2 (*ret/ptc2* papillary thyroid cancer oncogene) at Tyr586 in a phosphorylation-dependent manner, while both other LIM domains interact with several variants of PKC[11,31,43,44].

Confocal and immunofluorescent microscopy studies confirmed these data by showing that Enigma colocalized with RET/PTC2 at the cell periphery[43]. In addition, it was shown that the interaction of the second LIM domain of Enigma with RET/PTC2 is essential for the mitogenic activities of this protein[43].

The PDZ domain of Enigma, on the other hand, has been shown to bind to and colocalize with the C-terminal sequences of skeletal beta-tropomyosin (TPM2), which is a component of actin filaments[45]. Hence, resembling the other PDZ/LIM proteins, this suggests a role of Enigma in the vicinity of the sarcomere Z-line and at the boundary of the Z-line and I-band (Fig. 1B). A similar function had been described for ZASP. ZASP interacts with α-actinin through its PDZ domain, but is probably not dependent on it for translocation to the Z-lines of muscle. This observation is in line with the characteristics of the ZM domain in the ALP protein, which was found to be the primary structure involved in its translocation[14,33].

In contrast to the other PDZ/LIM genes, Enigma was also found to be able to up-regulate the expression of several bone morphogenetic proteins (BMP-2, BMP-4, BMP-6, BMP-7, and transforming growth factor-beta-1 [TGFβ1]), which suggests a role of Enigma in bone formation and metabolism[36,46]. The LIM domain was found dispensable for this function[38]. Still, as BMPs have been implicated in functions ranging from development of neural crest cells into neuronal phenotypes to giving direction to somite development through limb bud inhibition, the effects of Enigma might act on a larger scale than the above suggests[47].

Nakagawa et al. showed that ENH colocalizes with α-actinin in Z-disks and that the PDZ domain of ENH is necessary for this interaction[20]. It was further shown that ENH binds to protein kinase C-epsilon (PKC-ε) and the C-terminus of the N-type calcium channel alpha-1B subunit, and that ENH expression resulted in increased rapid and specific modulation of N-type calcium channels by PKC-ε[48]. In concert with these findings are the expression domains described for ENH in specific brain regions, namely, the cortex, hippocampus, hypothalamus, and the amygdala[48].

Important for the direct regulation of muscle contraction, force generation, and mechanical integrity is the regulation of PKC-mediated phosphorylation[49,50,51]. It has been shown for all three Enigma subfamily members (Enigma, ENH, and ZASP) that they are able to bind to PKC via their C-terminal LIM domains (Fig. 1B). Since they are able to bind to important sarcomere proteins, such as α-actinin and tropomyosin, via their PDZ domain, they are directly involved in targeting PKC to the sarcomere. Phosphorylation substrates of PKC in the sarcomere are, for example, troponin, vinculin, and ENH[31,51,52].

### Role in Development and Disease

Enigma, which has also been described as the LIM mineralization protein 1 (LMP1), has been shown to initiate membranous bone formation *in vitro* and *in vivo*[36]. Furthermore, Liu et al. demonstrated that transfected full-length *Enigma* induced bone nodule formation in rat calvarial osteoblasts[38]. Despite its colocalization with TPM2 at the Z-line and I-band, a definite functional role in the sarcomere has not been shown yet[45].

In contrast to Enigma, very limited information is available on the role of ENH in vertebrate development. The expression studies of Maeno-Hikichi et al., who found the ENH protein expressed in various regions of the brain, suggest a possible role in brain development[48]. In accordance, ENH expression levels were found to be significantly increased in all brain regions of patients with bipolar disorder, schizophrenia, and major depression[37,53]. It was further shown that different alleles of a specific ENH polymorphism bound differently to nuclear proteins, and the authors postulated a possible role of ENH in the susceptibility to schizophrenia[37].

Mutations in the *ZASP* gene have been associated with dilated cardiomyopathy (DCM) and DCM associated with isolated left ventricular noncompaction of the myocardium (INLVM) in humans[54,55]. The latter disease is generally characterized by a hypertrophic dilated left ventricle, ventricular dysfunction, and deep trabeculations. The presence of multiple mutations in the *ZASP* gene in patients with DCM and INLVM suggests that disruption of this gene is a common cause of left ventricular dysfunction and dilation. Recently, mutations in *ZASP* have been linked to a novel form of muscular dystrophy in humans[56].

*ZASP* has been described to be predominantly expressed in cardiac and skeletal muscles in mammals[22,23,24]. Importantly, *ZASP* ablation in mice was shown to be embryonic or perinatal lethal, most likely due to functional failure in multiple striated muscle types that displayed disorganized and fragmented Z-lines in skeletal and cardiac muscle[33]. This phenotype of the *ZASP* null mice suggested a role at maintaining Z-line structure, possibly by functioning as a scaffolding protein via its PDZ and LIM domains. Subsequent investigation showed that phenotypic mice could be rescued partially by a shorter splice form originating from *ZASP* lacking the LIM domains, but containing the PDZ domain and the ZM motif[57]. In zebrafish, *ZASP* knockdown led to severe heart and muscle defects. It was suggested that it plays a role downstream of Sonic Hedgehog in skeletal muscle development, in a late stage of somite development, when slow muscle fibers differentiate and migrate from the adaxial cells[34]. Similar to the mouse, a shorter splice form containing the PDZ, but lacking the LIM, domains was able to rescue the phenotype[34].

### THE ALP SUBFAMILY (ALP, ELFIN, MYSTIQUE, AND RIL)

The four mammalian ALP subfamily proteins — ALP, Elfin, Mystique, and RIL — are characterized by the presence of an N-terminal PDZ domain followed by a C-terminal LIM domain, and are postulated to play a role in actin anchorage in muscle as well as nonmuscle cells[19,58,59,60,61,62]. Expression of *Alp* in the mouse has been reported to be primarily in muscle cells, and it appears to have an essential role in the heart muscle[60,63,64]. Indeed, mice that lack *Alp* function develop cardiomyopathies[63,65]. The other ALP subfamily members — Elfin, Mystique, and RIL — are expressed more ubiquitously and are also found in several nonmuscle tissues and in epithelia[19,66,67,68]. *Elfin* has been described to be expressed during early heart development in the mouse[69]. Targeted gene silencing of *Mystique* in breast cancer cells with small interfering RNA (siRNA) suggested a role in the migratory capacity of epithelial cells[66], whereas RIL seems to function in neuronal signaling due to its involvement in AMPA (alpha-amino-3-hydroxy-5-methyl-4-isoxazole propionate) glutamate receptor recycling[70]. Additionally, *RIL* might play a role in tumor growth and progression[13].

#### Genomic Structure and Alternative Splicing

The smallest of the ALP subfamily genes, *RIL*, was the first to be discovered[13] and encompasses 7 exons in humans. The largest gene within this subfamily, *Mystique,* with 11 exons, is located at 8p21.1, a region that has been associated with ovarian and prostate cancers[71,72]. All genes within the ALP subfamily encode an N-terminal PDZ and a single C-terminal LIM domain (see Fig. 2A). At exon 5 (8 for *Mystique*), the coding sequence for a conserved ALP-like motif is present[16]. Similar to the Enigma subfamily gene *ZASP*, *ALP* and *Elfin* encode two and one ZM domain, respectively[14,15].

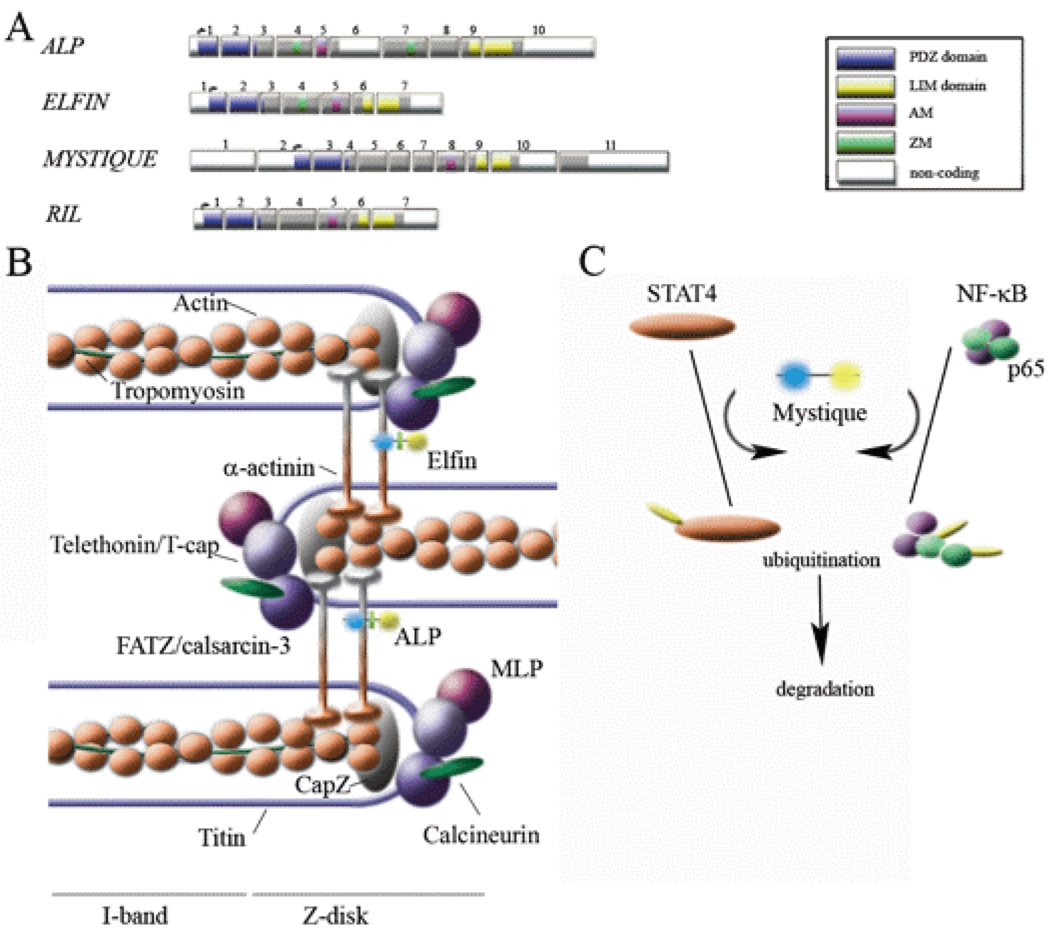

**FIGURE 2**. (A) The gene architectures of the ALP subfamily. The structures depicted are based on the following Ensembl references: *ALP* (ENSG00000154553), *ELFIN* (ENSG00000107438), *MYSTIQUE* (ENSG00000120913), and *RIL* (ENSG00000131435). The color-coding scheme is similar to that of Fig. 1, but unlike the Enigma subfamily, the ALP subfamily encodes an ALP-like motif (AM), which is indicated in purple. (B) Molecular interactions of the ALP subfamily. Shown is a simplified overview of the proteins in the sarcomere, highlighting the interactions mediated by the proteins of the ALP subfamily. The PDZ domains of the proteins in the ALP subfamily interact with α-actinin, which is bound to, for example, actin and titin. The interactions mediated by the LIM domains of the ALP subfamily proteins are unknown at present, but it is likely that they present docking sites for kinases. (C) E3 ligase activity of Mystique. Mystique not only functions in close association with the cytoskeleton, it has also been shown to function as an ubiquitin ligase. Its primary targets have so far been identified as the p65 subunit of NF-κB and both STAT-2 and STAT-4. It is not known where the ligating activity is located within the Mystique protein.

As shown for the Enigma subfamily, alternative splicing is used to generate a larger diversity of biologically active molecules within the ALP subfamily. To date, two full-length transcripts have been described for ALP (each having a different tissue-specific ZM motif), but an alternative termination site has been predicted in the zebrafish gene, which makes the existence of more splice variants very likely[14,73]. As to whether the different forms of ALP also perform different functions in the cell, it was recently observed that differentiation of myoblasts is accompanied by extensive switching between two ALP-derived splice forms[74].

The *RIL* gene has been shown to give rise to at least three splice products after transcription[58]. Three transcripts have been recognized for the Mystique-encoding gene and two that originate from the *Elfin* gene[66,73]. A conserved feature among these transcripts is that from each gene, a splice variant exists that only encodes the PDZ domain[66,73] and should, if translated, therefore lack all molecular interaction capabilities associated with the LIM domain (see below and Table 1).

### Molecular Interactions

The interactions of ALP with cytoskeletal proteins are believed to be mainly mediated via both its PDZ domain and ZM motif[14]. It has been demonstrated through mutation studies and inhibition experiments with synthetic peptides that the PDZ domain of Elfin and ALP is responsible for the binding to the C-terminal EF hands of α-actinin (more specifically to the amino acid motif ESDL)[14,60]. This site is close to the interaction site of α-actinin and titin in the sarcomere structure[75]. The ZM motif was found to bind the rod region of α-actinin[14]. So far, there has been one attempt to demonstrate the function of this interaction. In their studies, Klaavuniemi et al. showed that the binding of ALP to α-actinin does not interfere with the binding of α-actinin to F-actin directly, and they hypothesized that the stabilization of α-actinin by ALP depends on the possible interactions that α-actinin can make with F-actin[14]. This idea is in line with the observed actin patterns observed in muscle, which commonly are antiparallel in shape when they link opposing sarcomeres[76]. A plausible function of ALP (and possibly Elfin and ZASP, which also contain ZM motifs) may thus be the organization of actin filaments in striated muscle by orchestrating the possible cross-links between α-actinin and actin.

Similar to ALP, Elfin has been shown to interact with the EF hand region of α-actinin[62]. Elfin colocalizes with α-actinin in the Z-disks of myocardial sarcomeres (Fig. 2B), in particular in the vicinity of vinculin[62]. In nonmuscle cells, on the other hand, Elfin appears to interact with CLIK1 through its LIM domain, thereby acting as an adaptor for recruiting the CLIK1 kinase to actin stress fibers[77].

The *Mystique* gene is known to give rise to at least three splice forms in humans[66]. Apart from different expression patterns and different structures, these three Mystique variants appear also to bind to different proteins. For instance, Mystique variant 1 and variant 2 colocalize with proteins of the cytoskeleton, while Mystique variant 3 appears to be targeted to the nucleus[61]. In rodents, a Mystique variant is able to coprecipitate with α-actinin 2, αactinin 4, filamin, and myosin[61]. However, in total contrast to the other ALP subfamily proteins, Mystique has also been found in the nucleus, where it interacts with tyrosine-phosphorylated STAT4 (Signal Transducer and Activator of Transcription 4) and the p65 subunit of NF-κB[78,79]. It was further demonstrated that Mystique possesses ubiquitin E3 ligase activity, using both itself, the STAT proteins, or p65 as target[78,79] (Fig. 2C). A LIM domain mutation mutant was not able to show ubiquitination activity[78]. The newly discovered E3 ligase activity of Mystique has not been shown for any other of the PDZ and LIM domain–encoding proteins.

Analogous to Elfin and ALP, RIL has also been found to bind and colocalize with α-actinin, albeit more weakly than Elfin[67]. It might be hypothesized that this is a result of the absence of the ZM motif in RIL, which has been shown to be indispensable for proper α-actinin binding by ALP and ZASP[14,15]. Further investigation, however, has shown that the interaction between α-actinin and RIL is sufficient to stimulate the ability of α-actinin to bind to F-actin[67]. In addition to the interaction mentioned above, Van den Berk et al. showed that RIL interacts with the large submembranous protein tyrosine phosphatase PTP-BL through its LIM domain and its C-terminus, and that this interaction was mediated by one of the five PDZ domains present in this tyrosine phosphatase[80]. Interestingly, RIL was also shown to interact to AMPA glutamate receptor via its LIM domain, linking it to α-actinin via its PDZ domain for transport[70].

### Role in Development and Disease

The *ALP* gene was found to be primarily expressed in muscle tissue and ablation of *ALP* in mice caused embryonic chamber dysmorphogenesis, a failure of trabeculation and chamber dilation, eventually resulting in cardiomyopathy in adult mice[60,63,81]. However, ALP-deficient mice did not show an apparent phenotype in skeletal muscle[63,82]. These observations led to the hypothesis that ALP is redundant in murine muscle development and can be compensated by an alternative PDZ/LIM protein[82].

**TABLE 1**
**Synonyms, Gene Expression, Molecular Interactions, and Role in Development and Disease**

| Protein | Synonyms | Domain | Interaction | Complex | Expression | Development/ Disease | Ref. |
|---|---|---|---|---|---|---|---|
| ALP | PDLIM3 | PDZ | α-Actinin 2 | Sarcomeres | Cardiac and skeletal muscle; more ubiquitous in fish | Heart development, cardiomyopathies | [14,26,60,73] |
| | | ZM | α-Actinin 2 | | | | |
| | | LIM | — | | | | |
| Elfin | PDLIM1, CLIM 1, CLP 36 | PDZ | α-Actinin 1 and 4, PCMA | Sarcomeres, cytoskeleton | Cardiac and skeletal muscle, lungs, liver and other epithelial cells | | [19,59,62,68,69,73, 77,143] |
| | | ZM | — | | | | |
| | | LIM | α-Actinin 2, Clik1 | | | | |
| Enigma | PDLIM7, LMP-1 | PDZ<br>LIMs | β-Tropomyosin InsR, Ret/ptc2, PKCα, δ, ζ, APS | Sarcomeres, cytoskeleton | Skeletal muscle, bone | Bone morphogenesis | [38,43,44,45,144] |
| Enigma homolog | PDLIM5, ENH | PDZ | α-Actinin | Sarcomeres, cytoskeleton | Cardiac and skeletal muscle, brain (neurons) | Schizophrenia? | [20,41,42,48] |
| | | LIMs | PKC β, γ, ε, N-type $Ca^{2+}$ channels, LD2 | | | | |
| LIMK1 | LIM domain kinase 1 | LIMs | Kinase domain, PKC-γ, ζ, BMP7, p57, BMPR-II | Cytoskeleton, neuritic tips, nucleus | Nervous system (axons), lung, kidney, testis | Neurite extension, CNS development, Williams syndrome, cardiovascular disorders? Cancer, tumor-cell invasion, and metastasis. | [18,27,28,30,114, 145,146,147,148, 149] |
| | | PDZ | Nuclear export | | | | |
| | | Kinase | F-actin | | | | |
| LIMK2 | LIM domain kinase 2 | | — | Cytoskeleton, nucleus | Heart, brain, kidney, testis, lung | Testis development, neurite extension, cardiovascular disorders? Cancer, tumor-cell invasion, and metastasis. | [30,92,117,150,151] |
| LMO7 | LMO7, PCD1, FBXO20 | CH | α-Actinin | Adherens junctions | Heart, brain, liver, lung, skeletal muscle | Retinal degeneration, muscle degeneration, growth retardation, enhanced expression in tumors and linked to invasiveness | [25,130,131] |
| | | PDZ | Afadin | | | | |
| | | LIM | Emerin | | | | |
| Mystique | PDLIM2 | PDZ | α-Actinin | Nucleus, cytoskeleton | Heart, spleen, testis | Migration of breast cancer cells | [66,73] |
| | | LIM | — | | | | |
| RIL | PDLIM4 | PDZ | α-Actinin | Cytoskeleton | Brain | Neuronal development? Bone development? Tumor progression | [58,73,80,88,152] |
| | | LIM | AMPA receptor, PTP-BL | | | | |
| ZASP | PDLIM6, Cypher, Oracle, LDB3 | PDZ | α-Actinin 2 | Sarcomeres | Cardiac and skeletal muscle | Skeletal and cardiac muscle development, muscular and cardiomyopathies | [15,23,24] |
| | | ZM | α-Actinin 2 | | | | |
| | | LIMs | PKC α, β, γ, δ, ε, ζ | | | | |

*Elfin* expression in mice was found in the heart, lungs, liver, skin, and other epithelia, and, to a lesser extent, in skeletal muscle and the brain[19,59,67]. However, no important developmental role has been assigned to Elfin yet.

Mystique has been shown to be expressed and to associate with α-actinin in the cornea of the rat's eye and in the lungs of mice[61,66]. Knockout *Mystique* mice appear healthy and have normal numbers of lymphocyte subsets[78]. Consistent with the finding that Mystique is able to promote NF-κB degradation, PDLIM2–/– cells produced more IL6 and IL12p40 in response to CpG and lipopolysaccharides (LPS), well-known inducers of Toll-like receptor (TLR)–mediated proinflammatory responses[79]. Indeed, PDLIM2–/– mice were more susceptible to LPS-induced shock than wild-type mice[79]. Thus Mystique might play an important role in the immune system. Interestingly, overexpression of Mystique 2 in MCF-7 cells suppressed colony formation in soft agar and enhanced cell adhesion to collagen and fibronectin.

Point mutation of either the PDZ or LIM domain reversed the effect of suppressed colony formation, while mutation of the PDZ domain alone was sufficient to abolish enhanced adhesion. Knockdown of Mystique 2 with small interfering RNA abrogated both adhesion and migration in MCF10A and MCF-7 cells. Taken together, the data suggested that Mystique is located at the actin cytoskeleton and is necessary for the migratory capacity of epithelial cells. Additionally, it was observed that unlike normal transformed cells, the *mystique* knockdown cells migrated as sheets rather than individual cells. This suggests that *mystique* might also be crucially involved in tissues and at developmental stages where multi-cell migration has to be orchestrated, such as during wound repair[83,84].

The RIL protein–encoding gene was initially discovered in a search for candidate tumor suppressor genes in rat fibroblasts[13]. The human ortholog was identified on the chromosomal region 5q31.1, a cytokine cluster–containing region often having deletions in patients with myelodysplasia (MDP) and acute myeloid leukemia (AML)[58,85]. More recently, it was shown that in 70% (55 of 79) of tested cancer cell lines, the RIL-encoding gene was silenced by methylation[86,87]. A link between methylation and tumorigenesis was found in 60% of primary tumors tested and prostate tumors[86,87]. Interestingly, a single genomic variation in the *RIL* promoter has been linked with low bone mineral density (BMD) in Japanese women[88]. However, this is the only indication so far that RIL could be associated with the development of low bone mineral density.

### THE LIM DOMAIN KINASES (LIMK1 AND LIMK2)

The two LIM kinases share around 50% amino acid identity and hold the sequence information for two LIM domains, a single PDZ domain, and a tyrosine kinase domain. The order of the PDZ and LIM domains in these proteins is reversed as compared to the other PDZ/LIM proteins. The LIM kinases have their LIM domains positioned at the N-terminus.

However, their contributing function to the overall protein appears similar to the other PDZ/LIM proteins, and helps both LIMK1 and LIMK2 to play important roles in actin cytoskeleton organization. The LIM kinases have been linked to neural, reproductive, and cardiovascular disorders, and, more recently, to the progression of cancer[18,21,29].

#### Genomic Structure and Alternative Splicing

The human *LIMK1* gene maps to 7q11.23[12,89,90], while *LIMK2* was found to reside at 22q12[91]. The genomic structure of the two LIM kinase genes within and among species seems to be relatively well conserved[30,92,93,94]. Sixteen exons have been reported for the LIMK1 encoding genes to date, while up to 19 were identified for the LIMK2-encoding gene[30] (see Fig. 3A).

Over the years, several splice forms have been found to exist, which, considering their domain organization, might have noncanonical roles. The murine testis, for example, contains a specific LIMK2 splice variant, denoted LIMK2t, that lacks a significant portion of the transcript, since coding information for both LIM domains and a small part of the PDZ domain is absent[95,96]. Besides this testis-specific splice form and a full-length transcript (LIMK2a), several other tissue-specific LIMK2 transcripts have been described in mammals[91,93,94,96,97]. Examples are LIMK2b, which contains only three-quarters of the LIM-encoding region, and LIMK2c, which contains an insert in the kinase domain[94,96]. A transcript without the kinase domain has also been reported[98,99]. For LIMK2, different transcripts have been shown to have apparently their own tissue-specific promoters[93]. In line with this observation, it was recently shown for both LIMKs that both PDZ and LIM domains have overlapping expression patterns, but also specific expression domains[30]. In addition to the utilization of tissue-specific promoters, the LIMK transcripts are possibly also subject to tissue-specific splice regulations.

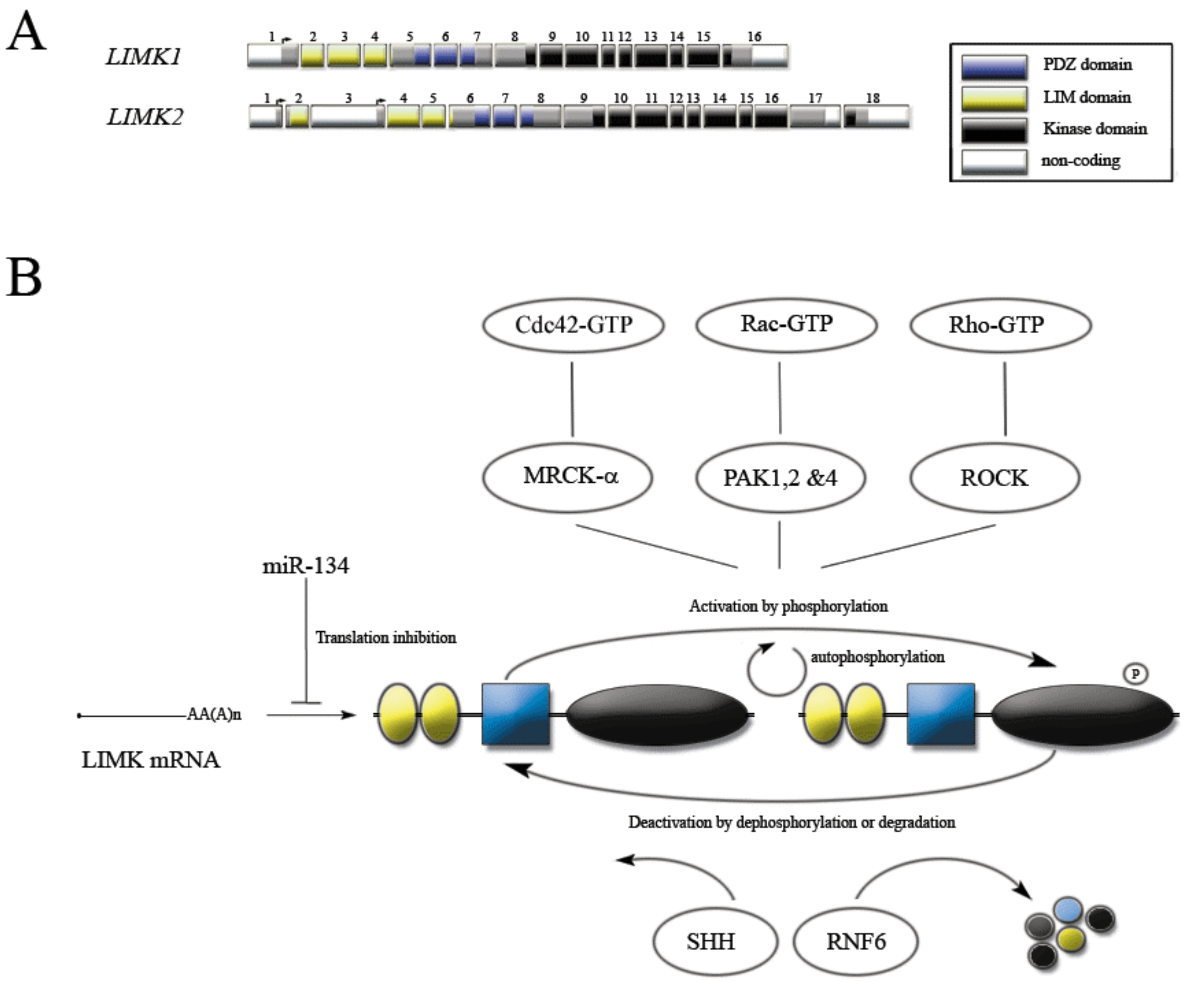

**FIGURE 3.** (A) The gene structures of the LIM domain kinases. Shown is an overview of the LIMK gene structures as harbored by the human genome. Structures are based on Ensembl references: *LIMK1* (ENSG00000106683, OTTHUMG00000023448) and *LIMK2* (ENSG00000182541). The PDZ and LIM domains are colored blue and yellow, respectively. The functionally important kinase domain is encoded by nine exons. An alternative transcription initiation site is also indicated for the *LIMK2* presenting structure. (B) Regulation of LIMK activity. LIMK activity and expression are highly regulated processes. First, translation of the LIMK-encoding mRNA may be reduced through microRNA miR-134. Phosphorylation and activation of LIMK can be mediated via three different pathways, all including members of the Rho GTPase family. Phosphorylation may also be a result of a transinteraction of two LIM kinases *in vitro*. Finally, inactivation of LIMK may result from dephosphorylation by Slingshot (SHH) or degradation induced by RNF-6.

### Molecular Interactions

Several LIMK interacting proteins have been identified over the years. The LIM kinases are best known for their involvement in deactivation of cofilins (the general term for the protein family containing cofilin1, cofilin2, and destrin [ADF]), which ultimately results in reorganization of the actin cytoskeleton and cell movement[18,21]. Cofilin mediates lamellipodium extension and polarized cell migration by accelerating actin filament dynamics at the leading edge of migrating cells, while it is also localized at the tips of growth cones in root ganglia neurons[100]. The cofilin mechanism is canonically inactivated by LIMK1-mediated phosphorylation of cofilin and it can be reactivated again by cofilin phosphatase Slingshot (SSH)-1L[101,102]. The inactivation of cofilin by LIMK is mediated by a phosphorylation of Serine 3, whereas the LIMK protein itself is activated by ROCK and deactivated by LATS1[29,103,104,105] (Fig. 3B). Recently, the transcription factors cAMP response element–binding protein (CREB) and Nurr1 were identified as possible additional substrates for the LIMK proteins in neurons[106,107].

Activation of LIMK1 may also be triggered by its binding to BMP7. This pathway, which needs both LIM domains of LIMK1, may function as an alternative to the SMAD-dependent pathway that links the BMP receptor regulation to actin dynamics[108]. Additional, though indirect, control over LIMK activity seems to come from Semaphorin 3A (Sema3A), a secreted glycoprotein involved in neuronal growth cone collapse, and fibrillar amyloid beta[109,110,111]. Moreover, cleavage of LIMK1 may occur at a caspase 3 type recognition site (a.a. 237-240; the C-terminal end of the PDZ domain), a site that is not conserved in LIMK2[112] or after polyubiquitination by Rnf6, which is highly expressed in developing neurons[113]. LIMK has also been shown to autophosphorylate *in vitro*[89].

Besides their functions in the cytoplasm, a function in the nucleus has been suggested for LIMK1. Nuclear import appears to be positively regulated by binding to p57 via the LIM domains and this interaction does not influence the kinase activity of the LIMK1 protein[114]. Phosphorylation on residues outside the activation sites of the kinase domain by PKC has been reported to prevent this nuclear import.

### Role in Development and Disease

Given the importance of the cytoskeleton for most cellular functions, e.g., motility, morphology, and internalization, it is not surprising that the LIM kinases are ubiquitously expressed during development, and that disruption of normal LIMK expression and/or signaling is associated with numerous disorders. For example, abnormal expression of LIMK1 in humans has been associated with Williams syndrome (a severe mental disorder with profound deficits in visuospatial cognition) and Alzheimer's disease[110,115]. In mice, ablation of LIMK1 leads to abnormalities in dendritic spine morphology and in synaptic function[116]. In contrast to *Limk1* null mice, *Limk2* knockout mice do not exhibit any impaired neuronal morphology or other phenotypic abnormalities in postnatal growth and development, except for impairment in spermatogenesis[117]. However, when a double knockout was applied, the neuronal phenotype appeared more pronounced than in the genetic deletion of *Limk1* alone[118].

In line with results of studies in mammals, the only LIM kinase present in *Drosophila melanogaster* was shown to be mainly involved in olfactory and neuromuscular development[119]. The importance of LIMK activity during development of the nervous system was recently further illustrated by the presence of a control mechanism, involving microRNA modulation (miR-134 blocked translation) of translation and proteasome-mediated degradation of LIMK1[113,120].

Motility of the neuronal growth cone is regulated by axon guidance molecules and their plasma membrane receptors. The secreted glycoprotein Sema3A functions as a negative regulator or repellant, inducing growth cone collapse at sites where it is bound by plexins and the neuropilin coreceptor, via a signaling pathway that involves Rac1[121,122,123]. A target molecule for this GTPase is LIMK, which has indeed been shown to be involved in growth cone motility and neurite extension, and to be a downstream component of Sema3A-induced growth cone collapse[111,121,124,125,126]. It is tempting to hypothesize that some of the observed neuronal disorders in LIMK knockdown/out experiments result from deregulation of the link between semaphorin-mediated neuronal guidance and LIMK activity in the cell. In addition to Sema3A, BMP and NGF have been shown to modulate LIMK activity and subsequently growth cone motility and morphology as well[125,127,128]. Taken together, all these results suggest that a tight regulation of cofilin activity through control of phosphorylation status via LIMK and SSH is critical for neurite extension and, subsequently, nervous system development, and that the LIMKs cannot be missed in helping to orchestrate the actin filament assembly at the tip of the growth cone. One might even go further and hypothesize that the LIMKs act as ultimate integrators of the two signaling pathways (i.e., Sema3A and BMP/NGF) and "decide" the fate of the growth cone and neuronal connections made during development.

### LIM DOMAIN ONLY 7

The LIM-only protein 7 (LMO7, PCD1, FBXO20) gene is by far the longest of the PDZ/LIM genes and encodes not only a PDZ and a C-terminal LIM domain, but also another protein interaction domain, a Calponin Homology domain. The presence of three different domains makes the original names ("LIM only" and "F-Box only" protein) to some extent ambiguous (a good alternative name could be PDLIM8). LMO7 appears to be ubiquitously expressed, although tissue-specific splice forms might exist. With regard to its role and function, studies have shown that LMO7 may be up-regulated in human tumors originating from the colon, breast, liver, lung, pancreas, stomach, and prostate. Additionally, LMO7 appears to be an important candidate for intriguing roles in embryonic muscle development[129], but also to regulate *emerin* expression in the nucleus and adherens junction integrity via linkage of the nectin-afadin and E-cadherin/catenin systems[25,130].

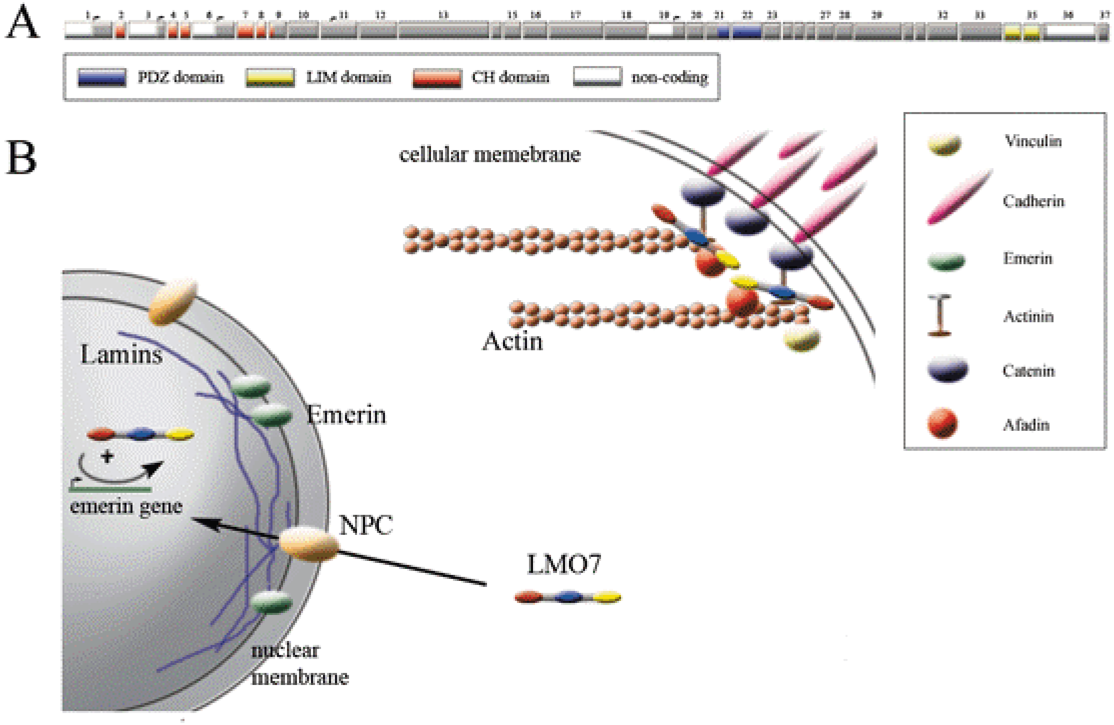

**FIGURE 4**. The gene architecture and molecular interactions of LIM domain only 7. (A) Depicted is the LMO7 gene structure as based on the Ensembl data (ENSG00000136153). In red, the exons encoding the CH domain are indicated. The exons shaded blue and yellow harbor the genetic information for both the PDZ and LIM domain, respectively. The gene structure shown also displays several alternative transcription initiation sites. (B) Shown is a summary of the interactions known to involve the LMO7 protein. Upon translocation to the nucleus, LMO7 is able to up-regulate *emerin* expression. Additionally, several other muscle-specific proteins may become regulated via a similar mechanism[130].

#### Genomic Structure and Alternative Splicing

The human *LMO7* gene maps to chromosome 13q21-q22, contains at least 27 exons (34 have been predicted), spans more than 238 kb, and encodes a protein of 1349 amino acids[131]. The most recent Ensembl exon organization data (depicted in Fig. 4A) suggests that the most N-terminal–conserved interaction domain, the CH domain, is encoded by exons 4, 5, and 7, and exon 6 contains an alternative start site. LMO7 exons 19 and 20 give rise to a PDZ domain, while the third- and second-to-last exons, 32 and 33, together form a single LIM domain.

To date, three splice forms have been described for human *LMO7* in addition to the full-length transcript. Two of these have been identified to lack a portion or the whole LIM domain, and appeared to be brain specific[132]. Another transcript appeared to be longer than the most ubiquitous splice variant and could only be isolated from skeletal muscle[132]. Two additional splice forms from rat tissue, termed LMO7b and LMO7s, have also been reported with the former one lacking a substantial region between the CH and PDZ domains[25,133], which contains the postulated F-box of LMO7[133,134]. Genescan predictions and EST databases have further predicted several additional splice forms, including some transcribed from alternative promoter regions.

### Molecular Interactions

All three domains found in LMO7 — the LIM domain, the CH domain, and the PDZ domain — are acknowledged protein interaction domains. Recently, it was shown that LMO7 binds to two F-actin binding proteins, α-actinin and afadin, via its PDZ and LIM domain, respectively[25].

It was confirmed by immunoprecipitation assays that these interactions linked LMO7 to the afadin-nectin and E-cadherin/catenin systems. Interestingly, the CH domain has been predicted to directly bind to actin[17].

Very recently, LMO7 was found to be involved in the regulation of transcription and protein function of emerin, a nuclear membrane protein. Additionally, LMO7 appeared to be involved in the transcriptional regulation of several muscle-relevant genes, including *emerin* via a feedback mechanism[130] (Fig. 4B).

### Role in Development and Disease

Human and mouse *LMO7* are candidate genes for breast cancer development in the human chromosomal region 13q21-q22 and for embryonic lethality in the mouse Ednrbs-1Acrg deletion, respectively[129,131,135]. In mice, an 800-kb deletion affecting the genes for *LMO7* and Uchl3 was lethal between birth and weaning for 40% of homozygotes, and led to retinal degeneration, muscular degeneration, and growth retardation in the surviving homozygotes[129]. Furthermore, LMO7 was found to be up-regulated in several human tumors[136,137] and had been linked to lymph node metastasis in breast cancer[138]. In line with the latter finding, a recent study showed that TGFβ1 induces LMO7 expression while enhancing cell invasiveness[133].

The tissue distribution of LMO7 mRNA has been investigated by northern blot analysis and RT-PCR. LMO7 expression in adult humans was found prominently in lung, skeletal muscle, and kidney, and weaker expression was detected in thymus, prostate, testis, colon, spinal cord, adrenal gland, placenta, and liver tissue[131]. Additional expression was described in adult brain and pancreas tissue[136]. Expression in fetal tissue was found to be high in lung and heart, and very weak in brain, kidney, and liver[132]. Microarray and semi-quantitative RT-PCR studies of mouse hematopoietic cell lines showed that LMO7 mRNA was absent from neuroblasts and T lymphocytes[139]. In rat tissues, protein expression was detected in heart, lung, kidney, and small intestine, and cross-reactive bands were also detected in the brain[25].

## PERSPECTIVES

### Development and Cancer

Cell movements and cell migration are not only important during normal development, but are also critical steps in tumor invasion and metastasis. The actin cytoskeleton, which is very important for directed cell movements through the construction of protrusions (in form of lamellipodia and filopodia), plays important roles in development (e.g., heart development[140]) and is further tightly linked to cancer cell metastasis. Indeed, reorganization of the actin cytoskeleton is the primary mechanism of cell motility and is essential for most types of cell migration. It is, therefore, not unexpected that the PDZ/LIM genes, which are all linked to the actin cytoskeleton, have important roles in development and cancer (see Table 1). Fig. 5A summarizes the current knowledge on the involvement of the LIMK in cellular movements. The question of how some of the other PDZ/LIM genes are involved in cell motility still waits to be answered. At least LMO7 and Mystique have clearly been linked to cell migration and cancer (Fig. 5B middle) whereas RIL's role in cancer could be through increasing the cellular growth rate (Fig. 5B bottom). The regulation of cancer cell mobility by Mystique and LMO7 could be related to actin cytoskeleton reorganization, but a different mechanism could also be responsible. This remains an important issue for the future.

### Skeletal and Cardiac Muscle Development

During vertebrate embryogenesis, skeletal muscle cell differentiation is coordinated, spatially and temporally, through cell-cell signaling between the neural tube and newly forming somites. The interactions that take place between the muscle precursor cells generally result in an ordered series of changes in gene expression, which bring about the synthesis of myogenic regulatory factors, synthesis, and assembly of muscle proteins into myofibrils and isoform changes of the major contractile proteins. Taking into account the vast number of interactions that have to take place for proper organization of the proteins in the myofibrils and Z-bands, keeping all these proteins in their proper position is essential. A module allowing for interactions via both a PDZ and LIM domain appears to be crucial for organizing these complexes, and many of the PDZ/LIM-encoding proteins have been implicated here.

Most PDZ and LIM domain–encoding proteins have been demonstrated to interact with α-actinin via their PDZ domain. Specific functions have been found for ALP and ZASP, which target to sarcomeres via their ZM and PDZ domain, and help here to organize actin/α-actinin binding. Both ALP and ZASP have been linked to heart development, whereas ZASP has been shown to be important in skeletal muscle development. For the other PDZ/LIM genes, a role in these processes has not yet been demonstrated, but is to be expected for at least some of them. Our preliminary data already suggest a role for LMO7 in heart development in the zebrafish.

Many PDZ/LIM genes seem to function in close association with bone morphogenic proteins, which are important players in development. The Enigma protein has been shown to up-regulate its expression, LIMK1 has been shown to bind the BMP type II receptor, LIMK2 responds to TGFβ1, and the LIMK-SSH balance is under control of BMP-7[127,128,141]. Additionally, ZASP appears to be under the control of Sonic Hedgehog, which has often been shown to cooperate with BMP molecules to control cell fates in development[34,142]. How and what the extent of these mechanisms is for developmental patterning, however, is still relatively unknown.

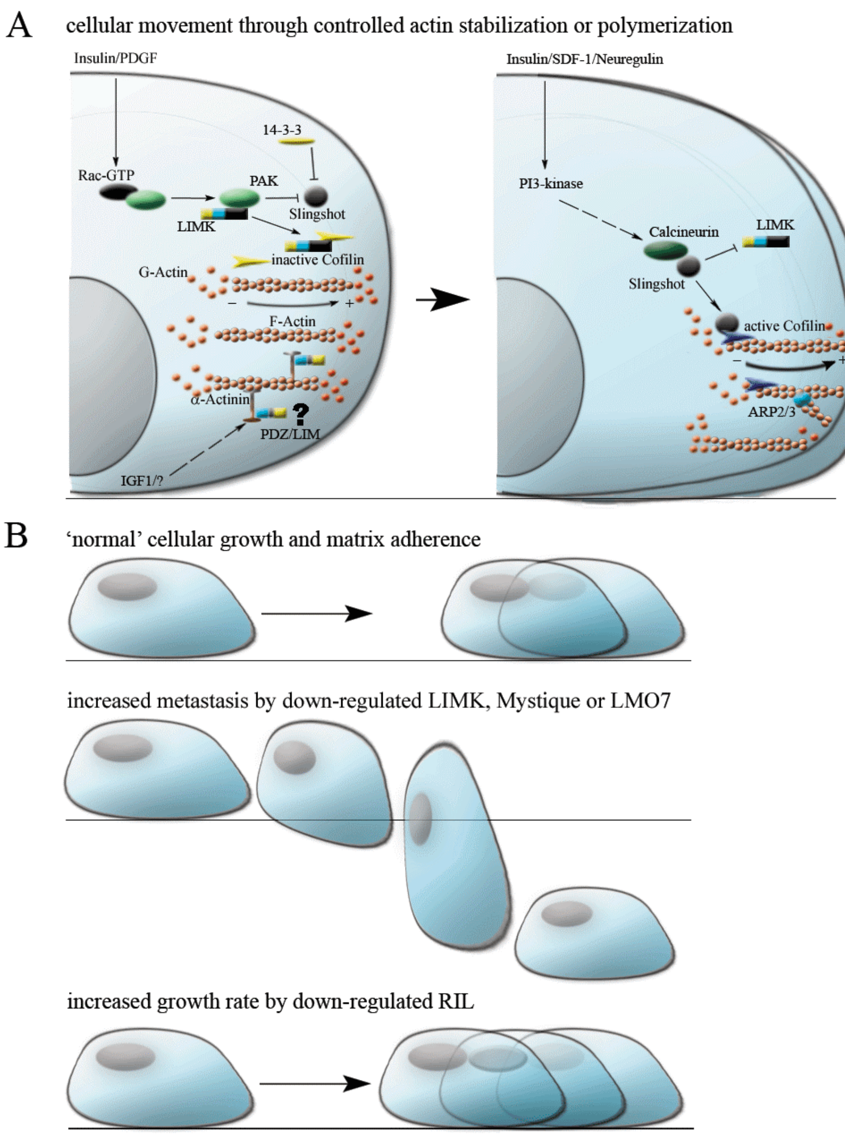

**FIGURE 5.** Involvement of PDZ/LIM proteins in cell motility and cancer. The PDZ/LIM protein LIMK is activated through growth factor signaling via small G-proteins (Cdc42/Rac/Rho; see also Fig. 3B) and, in turn, phosphorylates cofilin. This will render cofilin inactive and the actin cytoskeleton largely in treadmilling. When cell movements have to be initiated, however, Slingshot is activated. It will activate cofilin through dephosphorylation, thereby inducing actin destabilization at the minus ends, but an increased polymerization at the plus ends. It will also inactivate LIMK activity and is itself inhibited by both PAK and 14-3-3. Overall, the spatial and temporal regulation of cofilin activity by LIM kinase and Slingshot is critical for cell movements and directional cell migration, and the balance of these processes determines motility. How the other PDZ/LIM proteins lacking the kinase domain facilitate cell movements is not known. Although the mechanism is still elusive, it has clearly been demonstrated that Mystique and LMO7 are important in cell migration. (B) The upper panel shows the result of reduced LIMK activity in the cell. The same result has been noted for Mystique, but the mechanism is still unknown.

In this review, we describe the many commonalities as well as the specific differences not only within the group of PDZ/LIM genes, but also among the different splice forms of their individual members. Given that there are extra domains, in the case of the LIMK, even with enzymatic activity, it is clear that this will result in major functional differences. For LMO7, its large size in comparison to the other PDZ/LIM genes probably also contributes to some major functional differences. This angle ultimately leads to three central questions: How and why came this combination of the PDZ/LIM domains into existence? Why did it arise in ten different genes? An additional important question is: Are the commonalities among these genes (e.g., the localization at the actin cytoskeleton) mainly the result of the shared PDZ/LIM module? The answers to any of these questions are further complicated by the many different splice forms found for the PDZ/LIM genes and by possible redundancies between the different members. ZASP knockdown in both zebrafish and mouse embryos, for example, could be phenotypically rescued by expressing a splice form encoding the PDZ and ZM domain only[34]. So why would the gene encode a LIM domain if it is seemingly not essential, or does this imply that it is essential only at specific locations? Also, a ZASP knockout does not have as severe an effect in mice as the knockdown in zebrafish showed. Is there a PDZ/LIM gene in the mouse that compensates here for loss of Cypher (ZASP)? At present we can only speculate about these redundancies. Knockdown of several PDZ/LIM family members results only in relatively confined developmental effects (e.g., ALP, LIMKs; see previous sections). It would be interesting, in future experiments, to look at (invertebrate) species that encode only one or two of the PDZ/LIM genes, and to study the possible redundancies in PDZ/LIM functions. Furthermore, it will be interesting to learn more about the individual differences between the PDZ- and LIM-encoding proteins, and their role in skeletal and cardiac muscle development and function in general. Studying this group of genes will help to pin-point their roles in development and disease, and may further our insight into the important role of the actin cytoskeleton and its associated proteins in vertebrate development.

## References

1. Harris, B.Z. and Lim, W.A. (2001) Mechanism and role of PDZ domains in signaling complex assembly. *J. Cell Sci*. **114,** 3219–3231.
2. Cho, K.-O., Hunt, C.A., and Kennedy, M.B. (1992) The rat brain postsynaptic density fraction contains a homolog of the drosophila discs-large tumor suppressor protein. *Neuron* **9,** 929–942.
3. Woods, D.F. and Bryant, P.J. (1991) The discs-large tumor suppressor gene of Drosophila encodes a guanylate kinase homolog localized at septate junctions. *Cell* **66,** 451–464.
4. Itoh, M., Nagafuchi, A., Yonemura, S., Kitani-Yasuda, T., Tsukita, S., and Tsukita, S. (1993) The 220-kD protein colocalizing with cadherins in non-epithelial cells is identical to ZO-1, a tight junction-associated protein in epithelial cells: cDNA cloning and immunoelectron microscopy. *J. Cell Biol.* **121,** 491–502.
5. Ponting, C.P. (1997) Evidence for PDZ domains in bacteria, yeast, and plants. *Protein Sci.* **6**, 464–468.
6. Ponting, C.P., Phillips, C., Davies, K.E., and Blake, D.J. (1997) PDZ domains: targeting signalling molecules to sub-membranous sites. *Bioessays* **19,** 469–479.
7. Jelen, F., Oleksy, A., Smietana, K., and Otlewski, J. (2003) PDZ domains - common players in the cell signaling. *Acta Biochim. Pol.* **50,** 985–1017.
8. Bach, I. (2000) The LIM domain: regulation by association. *Mech. Dev*. **91,** 5–17.
9. Dawid, I.B., Breen, J.J., and Toyama, R. (1998) LIM domains: multiple roles as adapters and functional modifiers in protein interactions. *Trends Genet*. **14,** 156–162.
10. Kadrmas, J.L. and Beckerle, M.C. (2004) The LIM domain: from the cytoskeleton to the nucleus. *Nat. Rev. Mol. Cell Biol*. **5,** 920–931.
11. Wu, R. and Gill, G.N. (1994) LIM domain recognition of a tyrosine-containing tight turn. *J. Biol. Chem.* **269,** 25085–25090.
12. Mizuno, K., Okano, I., Ohashi, K., Nunoue, K., Kuma, K., Miyata, T., and Nakamura, T. (1994) Identification of a human cDNA encoding a novel protein kinase with two repeats of the LIM/double zinc finger motif. *Oncogene* **9,** 1605–1612.
13. Kiess, M., Scharm, B., Aguzzi, A., Hajnal, A., Klemenz, R., Schwarte-Waldhoff, I., and Schafer, R. (1995) Expression of ril, a novel LIM domain gene, is down-regulated in Hras-transformed cells and restored in phenotypic revertants. *Oncogene* **10,** 61–68.
14. Klaavuniemi, T., Kelloniemi, A., and Ylanne, J. (2004) The ZASP-like motif in actinin-associated LIM protein is required for interaction with the {alpha}-actinin rod and for targeting to the muscle Z-line. *J. Biol. Chem*. **279,** 26402–26410.
15. Klaavuniemi, T. and Ylanne, J. (2006) Zasp/Cypher internal ZM-motif containing fragments are sufficient to co-localize with α-actinin—analysis of patient mutations. *Exp. Cell Res*. **312,** 1299–1311.
16. te Velthuis, A.J.W., Isogai, T., Gerrits, L., and Bagowski, C.P. (2007) Insights into the molecular evolution of the PDZ-LIM family and identification of a novel conserved protein motif. *PLoS ONE* **2,** e189.
17. Lehman, W., Craig, R., Kendrick-Jones, J., and Sutherland-Smith, A. (2004) An open or closed case for the conformation of calponin homology domains on F-actin? *J. Muscle Res. Cell Motil.* **25,** 351–358.
18. Yang, N., Higuchi, O., Ohashi, K., Nagata, K., Wada, A., Kangawa, K., Nishida, E., and Mizuno, K. (1998) Cofilin phosphorylation by LIM-kinase 1 and its role in Rac-mediated actin reorganization. *Nature* **393,** 809–812.
19. Vallenius, T., Luukko, K., and Makela, T.P. (2000) CLP-36 PDZ-LIM protein associates with nonmuscle alphaactinin-1 and alpha-actinin-4. *J. Biol. Chem*. **275,** 11100–11105.

20. Nakagawa, N., Hoshijima, M., Oyasu, M., Saito, N., Tanizawa, K., and Kuroda, S. (2000) ENH, containing PDZ and LIM domains, heart/skeletal muscle-specific protein, associates with cytoskeletal proteins through the PDZ domain. *Biochem. Biophys. Res. Commun*. **272,** 505.
21. Arber, S., Barbayannis, F.A., Hanser, H., Schneider, C., Stanyon, C.A., Bernard, O., and Caroni, P. (1998) Regulation of actin dynamics through phosphorylation of cofilin by LIM-kinase. *Nature* **393,** 805–809.
22. Faulkner, G., Pallavicini, A., Formentin, E., Comelli, A., Ievolella, C., Trevisan, S., Bortoletto, G., Scannapieco, P., Salamon, M., Mouly, V., Valle, G., and Lanfranchi, G. (1999) ZASP: a new Z-band alternatively spliced PDZ-motif protein. *J. Cell Biol*. **146,** 465–476.
23. Zhou, Q., Ruiz-Lozano, P., Martone, M.E., and Chen, J. (1999) Cypher, a striated muscle-restricted PDZ and LIM domain-containing protein, binds to alpha-actinin-2 and protein kinase C. *J. Biol. Chem*. **274,** 19807–19813.
24. Passier, R., Richardson, J.A., and Olson, E.N. (2000) Oracle, a novel PDZ-LIM domain protein expressed in heart and skeletal muscle. *Mech. Dev*. **92,** 277–284.
25. Ooshio, T., Irie, K., Morimoto, K., Fukuhara, A., Imai, T., and Takai, Y. (2004) Involvement of LMO7 in the association of two cell-cell adhesion molecules, nectin and Ecadherin, through afadin and alpha-actinin in epithelial cells. *J. Biol. Chem*. **279,** 31365–31373.
26. Andersen, O., Ostbye, T.K., Gabestad, I., Nielsen, C., Bardal, T., and Galloway, T.F. (2004) Molecular characterization of a PDZ-LIM protein in Atlantic salmon (Salmo salar): a fish ortholog of the alpha-actinin-associated LIM-protein (ALP). *J. Muscle Res. Cell Motil*. **25,** 61.
27. Gorovoy, M., Niu, J., Bernard, O., Profirovic, J., Minshall, R., Neamu, R., and Voyno-Yasenetskaya, T. (2005) LIM kinase 1 coordinates microtubule stability and actin polymerization in human endothelial cells. *J. Biol. Chem*. **280,** 26533–26542.
28. Foletta, V.C., Moussi, N., Sarmiere, P.D., Bamburg, J.R., and Bernard, O. (2004) LIM kinase 1, a key regulator of actin dynamics, is widely expressed in embryonic and adult tissues. *Exp. Cell Res*. **294,** 392–405.
29. Sumi, T., Matsumoto, K., Takai, Y., and Nakamura, T. (1999) Cofilin phosphorylation and actin cytoskeletal dynamics regulated by Rho- and Cdc42-activated LIM-kinase 2. *J. Cell Biol*. **147,** 1519–1532.
30. Ott, E.B., Te Velthuis, A.J.W., and Bagowski, C.P. (2007) Comparative analysis of splice form-specific expression of LIM kinases during zebrafish development. *Gene Expr. Patterns* **7,** 620–629.
31. Kuroda, S., Toukunaga, C., Kiyohara, Y., Higuchi, O., Konischi, H., Mizuno, K., Gill, G.N., and Kikkawa, U. (1996) Protein-protein interaction of zinc finger LIM domains with protein kinase C. *J. Biol. Chem*. **271,** 31029–31032.
32. Ishikawa, K., Nagase, T., Suyama, M., Miyajima, N., Tanaka, A., Kotani, H., Nomura, N., and Ohara, O. (1998) Prediction of the coding sequences of unidentified human genes. X. The complete sequences of 100 new cDNA clones from brain which can code for large proteins in vitro. *DNA Res*. **5,** 169–176.
33. Zhou, Q., Chu, P.-H., Huang, C., Cheng, C.-F., Martone, M.E., Knoll, G., Shelton, G.D., Evans, S., and Chen, J. (2001) Ablation of Cypher, a PDZ-LIM domain Z-line protein, causes a severe form of congenital myopathy. *J. Cell Biol*. **155,** 605–612.
34. van der Meer, D.L.M., Marques, I.J., Leito, J.T.D., Besser, J., Bakkers, J., Schoonheere, E., and Bagowski, C.P. (2006) Zebrafish cypher is important for somite formation and heart development. *Dev. Biol*. **299,** 356–372.
35. Vatta, M., Mohapatra, B., Jimenez, S., Sanchez, X., Faulkner, G., Perles, Z., Sinagra, G., Lin, J.-H., Vu, T.M., and Zhou, Q. (2003) Mutations in Cypher/ZASP in patients with dilated cardiomyopathy and left ventricular non-compaction. *J. Am. Coll. Cardiol*. **42,** 2014–2027.
36. Boden, S.D., Liu, Y., Hair, G.A., Helms, J.A., Hu, D., Racine, M., Nanes, M.S., and Titus, L. (1998) LMP-1, a LIM-domain protein, mediates BMP-6 effects on bone formation. *Endocrinology* **139,** 5125–5134.
37. Horiuchi, Y., Arai, M., Niizato, K., Iritani, S., Noguchi, E., Ohtsuki, T., Koga, M., Kato, T., Itokawa, M., and Arinami, T. (2006) A polymorphism in the PDLIM5 gene associated with gene expression and schizophrenia. *Biol. Psychiatry* **59,** 434–439.
38. Liu, Y., Hair, G.A., Boden, S.D., Viggeswarapu, M., and Titus, L. (2002) Overexpressed LIM mineralization proteins do not require LIM domains to induce bone. *J. Bone Miner. Res*. **17,** 406–414.
39. Ueki, N., Seki, N., Yano, K., Masuho, Y., Saito, T., and Muramatsu, M. (1999) Isolation, tissue expression, and chromosomal assignment of a human LIM protein gene, showing homology to rat enigma homologue (ENH). *J. Hum. Genet*. **44,** 256–260.
40. Sierralta, J. and Mendoza, C. (2004) PDZ-containing proteins: alternative splicing as a source of functional diversity. *Brain Res. Brain Res. Rev*. **47,** 105–115.
41. Niederlander, N., Fayein, N.A., Auffray, C., and Pomies, P. (2004) Characterization of a new human isoform of the enigma homolog family specifically expressed in skeletal muscle. *Biochem. Biophys. Res. Commun*. **325,** 1304.
42. Lasorella, A. and Iavarone, A. (2006) The protein ENH is a cytoplasmic sequestration factor for Id2 in normal and tumor cells from the nervous system. *Proc. Natl. Acad. Sci. U. S. A.* **103(13),** 4976–4981.
43. Durick, K., Wu, R.-Y., Gill, G.N., and Taylor, S.S. (1996) Mitogenic signaling by Ret/ptc2 requires association with enigma via a LIM domain. *J. Biol. Chem*. **271,** 12691–12694.
44. Wu, R., Durick, K., Songyang, Z., Cantley, L.C., Taylor, S.S., and Gill, G.N. (1996) Specificity of LIM domain interactions with receptor tyrosine kinases. *J. Biol. Chem*. **271,** 15934–15941.
45. Guy, P.M., Kenny, D.A., and Gill, G.N. (1999) The PDZ domain of the LIM protein enigma binds to beta -

tropomyosin. *Mol. Biol. Cell* **10,** 1973–1984.
46. Minamide, A., Boden, S.D., Viggeswarapu, M., Hair, G.A., Oliver, C., and Titus, L. (2003) Mechanism of bone formation with gene transfer of the cDNA encoding for the intracellular protein LMP-1. *J. Bone Joint Surg. Am.* **85,** 1030–1039.
47. Chen, D., Zhao, M., and Mundy, G.R. (2004) Bone morphogenetic proteins. *Growth Factors* **22,** 233–241.
48. Maeno-Hikichi, Y., Chang, S., Matsumura, K., Lai, M., Lin, H., Nakagawa, N., Kuroda, S., and Zhang, J.F. (2003) A PKC[epsi]-ENH-channel complex specifically modulates N-type Ca2+ channels. *Nat. Neurosci.* **6,** 468.
49. Sakthivel, S., Finley, N.L., Rosevear, P.R., Lorenz, J.N., Gulick, J., Kim, S., VanBuren, P., Martin, L.A., and Robbins, J. (2005) In vivo and in vitro analysis of cardiac troponin I phosphorylation. *J. Biol. Chem.* **280,** 703–714.
50. Jideama, N.M., Noland, T.A., Jr., Raynor, R.L., Blobe, G.C., Fabbro, D., Kazanietz, M.G., Blumberg, P.M., Hannun, Y.A., and Kuo, J.F. (1996) Phosphorylation specificities of protein kinase C isozymes for bovine cardiac troponin I and troponin T and sites within these proteins and regulation of myofilament properties. *J. Biol. Chem.* **271,** 23277–23283.
51. Kobayashi, T., Dong, W.J., Burkart, E.M., Cheung, H.C., and Solaro, R.J. (2004) Effects of protein kinase C dependent phosphorylation and a familial hypertrophic cardiomyopathy-related mutation of cardiac troponin I on structural transition of troponin C and myofilament activation. *Biochemistry* **43,** 5996–6004.
52. Larsson, C. (2006) Protein kinase C and the regulation of the actin cytoskeleton. *Cell. Signal.* **18,** 276–284.
53. Kato, T., Iwayama, Y., Kakiuchi, C., Iwamoto, K., Yamada, K., Minabe, Y., Nakamura, K., Mori, N., Fujii, K., Nanko, S., and Yoshikawa, T. (2005) Gene expression and association analyses of LIM (PDLIM5) in bipolar disorder and schizophrenia *Mol. Psychiatry* **10,** 1045–1055.
54. Arimura, T., Hayashi, T., Terada, H., Lee, S.-Y., Zhou, Q., Takahashi, M., Ueda, K., Nouchi, T., Hohda, S., Shibutani, M., Hirose, M., Chen, J., Park, J.-E., Yasunami, M., Hayashi, H., and Kimura, A. (2004) A Cypher/ZASP mutation associated with dilated cardiomyopathy alters the binding affinity to protein kinase C. *J. Biol. Chem.* **279,** 6746–6752.
55. Vatta, M., Mohapatra, B., Jimenez, S., Sanchez, X., Faulkner, G., Perles, Z., Sinagra, G., Lin, J.-H., Vu, T.M., Zhou, Q., Bowles, K.R., Di Lenarda, A., Schimmenti, L., Fox, M., Chrisco, M.A., Murphy, R.T., McKenna, W., Elliott, P., Bowles, N.E., Chen, J., Valle, G., and Towbin, J.A. (2003) Mutations in Cypher/ZASP in patients with dilated cardiomyopathy and left ventricular non-compaction. *J. Am. Coll. Cardiol.* **42,** 2014–2027.
56. Selcen, D. and Engel, A.G. (2005) Mutations in ZASP define a novel form of muscular dystrophy in humans. *Ann. Neurol.* **57,** 269–276.
57. Huang, C., Zhou, Q., Liang, P., Hollander, M.S., Sheikh, F., Li, X., Greaser, M., Shelton, G.D., Evans, S., and Chen, J. (2003) Characterization and in vivo functional analysis of splice variants of Cypher. *J. Biol. Chem.* **278,** 7360–7365.
58. Bashirova, A.A., Markelov, M.L., Shlykova, T.V., Levshenkova, E.V., Alibaeva, R.A., and Frolova, E.I. (1998) The human RIL gene: mapping to human chromosome 5q31.1, genomic organization and alternative transcripts. *Gene* **210,** 239.
59. Wang, H., Harrison-Shostak, D.C., Lemasters, J.J., and Herman, B. (1995) Cloning of a rat cDNA encoding a novel LIM domain protein with high homology to rat RIL. *Gene* **165,** 267.
60. Xia, H., Winokur, S.T., Kuo, W.L., Altherr, M.R., and Bredt, D.S. (1997) Actinin-associated LIM protein: identification of a domain interaction between PDZ and spectrin-like repeat motifs. *J. Cell Biol.* **139,** 507–515.
61. Torrado, M., Senatorov, V.V., Trivedi, R., Fariss, R.N., and Tomarev, S.I. (2004) Pdlim2, a novel PDZ-LIM domain protein, interacts with {alpha}-actinins and filamin A. *Invest. Ophthalmol. Vis. Sci.* **45,** 3955–3963.
62. Kotaka, M., Kostin, S., Ngai, S., Chan, K., Lau, Y., Lee, S.M., Li, H., Ng, E.K., Schaper, J., Tsui, S.K., Fung, K., Lee, C., and Waye, M.M. (2000) Interaction of hCLIM1, an enigma family protein, with alpha-actinin 2. *J. Cell. Biochem.* **78,** 558–565.
63. Pashmforoush, M., Pomies, P., Peterson, K.L., Kubalak, S., Ross, J., Hefti, A., Aebi, U., Beckerle, M.C., and Chien, K.R. (2001) Adult mice deficient in actinin-associated LIM-domain protein reveal a developmental pathway for right ventricular cardiomyopathy. *Nat. Med.* **7,** 591.
64. Henderson, J.R., Pomies, P., Auffray, C., and Beckerle, M.C. (2003) ALP and MLP distribution during myofibrillogenesis in cultured cardiomyocytes. *Cell Motil. Cytoskeleton* **54,** 254–265.
65. Lorenzen-Schmidt, I., McCulloch, A.D., and Omens, J.H. (2005) Deficiency of actinin-associated LIM protein alters regional right ventricular function and hypertrophic remodeling. *Ann. Biomed. Eng.* **33,** 888–896.
66. Loughran, G., Healy, N.C., Kiely, P.A., Huigsloot, M., Kedersha, N.L., and O'Connor, R. (2005) Mystique is a new insulin-like growth factor-I-regulated PDZ-LIM domain protein that promotes cell attachment and migration and suppresses Anchorage-independent growth. *Mol. Biol. Cell* **16,** 1811–1822.
67. Vallenius, T., Scharm, B., Vesikansa, A., Luukko, K., Schafer, R., and Makela, T.P. (2004) The PDZ-LIM protein RIL modulates actin stress fiber turnover and enhances the association of [alpha]-actinin with F-actin. *Exp. Cell Res.* **293,** 117.
68. Kotaka, M., Ngai, S.M., Garcia-Barcelo, M., Tsui, S.K., Fung, K.P., Lee, C.Y., and Waye, M.M. (1999) Characterization of the human 36-kDa carboxyl terminal LIM domain protein (hCLIM1). *J. Cell. Biochem.* **72,** 279–285.
69. Kotaka, M., Lau, Y.M., Cheung, K.K., Lee, S.M., Li, H.Y., Chan, W.Y., Fung, K.P., Lee, C.Y., Waye, M.M., and

Tsui, S.K. (2001) Elfin is expressed during early heart development. *J. Cell. Biochem.* **83,** 463–472.
70. Schulz, T.W., Nakagawa, T., Licznerski, P., Pawlak, V., Kolleker, A., Rozov, A., Kim, J., Dittgen, T., Kohr, G., Sheng, M., Seeburg, P.H., and Osten, P. (2004) Actin/alpha-actinin-dependent transport of AMPA receptors in dendritic spines: role of the PDZ-LIM protein RIL. *J. Neurosci.* **24,** 8584–8594.
71. Brown, M.R., Chuaqui, R., Vocke, C.D., Berchuck, A., Middleton, L.P., Emmert-Buck, M.R., and Kohn, E.C. (1999) Allelic loss on chromosome arm 8p: analysis of sporadic epithelial ovarian tumors. *Gynecol. Oncol*. **74,** 98–102.
72. Swalwell, J.I., Vocke, C.D., Yang, Y., Walker, J.R., Grouse, L., Myers, S.H., Gillespie, J.W., Bostwick, D.G., Duray, P.H., Linehan, W.M., and Emmert-Buck, M.R. (2002) Determination of a minimal deletion interval on chromosome band 8p21 in sporadic prostate cancer. *Genes Chromosomes Cancer* **33,** 201–205.
73. te Velthuis, A.J.W., Ott, E.B., Marques, I.J., and Bagowski, C.P. (2007) Gene expression patterns of the ALP family during zebrafish development. *Gene Expr. Patterns* **7,** 297–305.
74. Pomies, P., Pashmforoush, M., Vegezzi, C., Chien, K.R., Auffray, C., and Beckerle, M.C. (2007) The cytoskeleton-associated PDZ-LIM protein, ALP, acts on serum response factor activity to regulate muscle differentiation. *Mol. Biol. Cell* **18,** 1723–1733.
75. Luther, P.K., Barry, J.S., and Squire, J.M. (2002) The three-dimensional structure of a vertebrate wide (slow muscle) Z-band: lessons on Z-band assembly. *J. Mol. Biol*. **315,** 9–20.
76. Atkinson, R.A., Joseph, C., Kelly, G., Muskett F W, Frenkiel, T.A., Nietlispach, D., and Pastore, A. (2001) Ca2+-independent binding of an EF-hand domain to a novel motif in the alpha-actinin-titin complex. *Nat. Struct. Biol*. **8,** 853–857.
77. Vallenius, T. and Makela, T.P. (2002) Clik1: a novel kinase targeted to actin stress fibers by the CLP-36 PDZ-LIM protein. *J. Cell Sci*. **115,** 2067–2073.
78. Tanaka, T., Soriano, M.A., and Grusby, M.J. (2005) SLIM is a nuclear ubiquitin E3 ligase that negatively regulates STAT signaling. *Immunity* **22,** 729–736.
79. Tanaka, T., Grusby, M.J., and Kaisho, T. (2007) PDLIM2-mediated termination of transcription factor NF-[kappa]B activation by intranuclear sequestration and degradation of the p65 subunit. *Nat. Immunol*. **8(6),** 584–591.
80. van den Berk, L.C.J., van Ham, M.A., te Lindert, M.M., Walma, T., Aelen, J., Vuister, G.W., and Hendriks, W.J.A.J. (2005) The interaction of PTP-BL PDZ domains with RIL: an enigmatic role for the RIL LIM domain. *Mol. Biol. Rep*. **31,** 203.
81. Pomies, P., Macalma, T., and Beckerle, M.C. (1999) Purification and characterization of an alpha-actinin-binding PDZ-LIM protein that is up-regulated during muscle differentiation. *J. Biol. Chem.* **274,** 29242–29250.
82. Jo, K., Rutten, B., Bunn, R.C., and Bredt, D.S. (2001) Actinin-associated LIM protein-deficient mice maintain normal development and structure of skeletal muscle. *Mol. Cell. Biol*. **21,** 1682–1687.
83. Martin, P. and Parkhurst, S.M. (2004) Parallels between tissue repair and embryo morphogenesis. *Development* **131,** 3021–3034.
84. Martin, P. (1997) Wound healing--aiming for perfect skin regeneration. *Science* **276,** 75–81.
85. Pedersen, B. (1996) Anatomy of the 5q- deletion: different sex ratios and deleted 5q bands in MDS and AML. *Leukemia* **10,** 1883–1890.
86. Boumber, Y.A., Kondo, Y., Chen, X., Shen, L., Gharibyan, V., Konishi, K., Estey, E., Kantarjian, H., Garcia-Manero, G., and Issa, J.P. (2007) RIL, a LIM gene on 5q31, is dilenced by methylation in cancer and sensitizes cancer cells to apoptosis. *Cancer Res*. **67,** 1997–2005.
87. Vanaja, D.K., Ballman, K.V., Morlan, B.W., Cheville, J.C., Neumann, R.M., Lieber, M.M., Tindall, D.J., and Young, C.Y.F. (2006) PDLIM4 repression by hypermethylation as a potential biomarker for prostate cancer. *Clin. Cancer Res*. **12,** 1128–1136.
88. Omasu, F., Ezura, Y., Kajita, M., Ishida, R., Kodaira, M., Yoshida, H., Suzuki, T., Hosoi, T., Inoue, S., Shiraki, M., Orimo, H., and Emi, M. (2003) Association of genetic variation of the RIL gene, encoding a PDZ-LIM domain protein and localized in 5q31.1, with low bone mineral density in adult Japanese women. *J. Hum. Genet*. **48,** 342.
89. Proschel, C., Blouin, M.J., Gutowski, N.J., Ludwig, R., and Noble, M. (1995) Limk1 is predominantly expressed in neural tissues and phosphorylates serine, threonine and tyrosine residues in vitro. *Oncogene* **11,** 1271–1281.
90. Mao, X., Jones, T.A., Williamson, J., Gutowski, N.J., Proschel, C., Noble, M., and Sheer, D. (1996) Assignment of the human and mouse LIM-kinase genes (LIMK1; Limk1) to chromosome bands 7q11.23 and 5G1, respectively, by in situ hybridization. *Cytogenet. Cell Genet*. **74,** 190–191.
91. Okano, I., Hiraoka, J., Otera, H., Nunoue, K., Ohashi, K., Iwashita, S., Hirai, M., and Mizuno, K. (1995) Identification and characterization of a novel family of serine/threonine kinases containing two N-terminal LIM motifs. *J. Biol. Chem*. **270,** 31321–31330.
92. Ikebe, C., Ohashi, K., Fujimori, T., Bernard, O., Noda, T., Robertson, E.J., and Mizuno, K. (1997) Mouse LIM-kinase 2 gene: cDNA cloning, genomic organization, and tissue-specific expression of two alternatively initiated transcripts. *Genomics* **46,** 504.
93. Nomoto, S., Tatematsu, Y., Takahashi, T., and Osada, H. (1999) Cloning and characterization of the alternative promoter regions of the human LIMK2 gene responsible for alternative transcripts with tissue-specific expression. *Gene* **236,** 259–271.
94. Koshimizu, U., Takahashi, H., Yoshida, M.C., and Nakamura, T. (1997) cDNA cloning, genomic organization, and chromosomal localization of the mouse LIM motif-containing kinase gene, Limk2. *Biochem. Biophys. Res. Commun.*

**241**, 243–250.

95. Takahashi, H., Koshimizu, U., and Nakamura, T. (1998) A novel transcript encoding truncated LIM kinase 2 is specifically expressed in male germ cells undergoing meiosis. *Biochem. Biophys. Res. Commun.* **249,** 138–145.
96. Ikebe, C., Ohashi, K., and Mizuno, K. (1998) Identification of testis-specific (Limk2t) and brain-specific (Limk2c) isoforms of mouse LIM-kinase 2 gene transcripts. *Biochem. Biophys. Res. Commun.* **246,** 307–312.
97. Osada, H., Hasada, K., Inazawa, J., Uchida, K., Ueda, R., Takahashi, T., and Takahashi, T. (1996) Subcellular localization and protein interaction of the human LIMK2 gene expressing alternative transcripts with tissue-specific regulation. *Biochem. Biophys. Res. Commun.* **13,** 582–589.
98. Edwards, D.C. and Gill, G.N. (1999) Structural features of LIM kinase that control effects on the actin cytoskeleton. *J. Biol. Chem.* **274,** 11352–11361.
99. Mori, T., Okano, I., Mizuno, K., Tohyama, M., and Wanaka, A. (1997) Comparison of tissue distribution of two novel serine/threonine kinase genes containing the LIM motif (LIMK-1 and LIMK-2) in the developing rat. *Mol. Brain Res.* **45,** 247–254.
100. Bamburg, J.R. and Bray, D. (1987) Distribution and cellular localization of actin depolymerizing factor. *J. Cell Biol.* **105,** 2817–2825.
101. Niwa, R., Nagata-Ohashi, K., Takeichi, M., Mizuno, K., and Uemura, T. (2002) Control of actin reorganization by Slingshot, a family of phosphatases that dephosphorylate ADF/cofilin. *Cell* **108,** 233.
102. Nishita, M., Tomizawa, C., Yamamoto, M., Horita, Y., Ohashi, K., and Mizuno, K. (2005) Spatial and temporal regulation of cofilin activity by LIM kinase and Slingshot is critical for directional cell migration. *J. Cell Biol.* **171,** 349–359.
103. Sumi, T., Matsumoto, K., and Nakamura, T. (2001) Specific activation of LIM kinase 2 via phosphorylation of threonine 505 by ROCK, a Rho-dependent protein kinase. *J. Biol. Chem.* **276,** 670–676.
104. Maekawa, M., Ishizaki, T., Boku, S., Watanabe, N., Fujita, A., Iwamatsu, A., Obinata, T., Ohashi, K., Mizuno, K., and Narumiya, S. (1999) Signaling from Rho to the actin cytoskeleton through protein kinases ROCK and LIM-kinase. *Science* **285,** 895–898.
105. Amano, T., Kaji, N., Ohashi, K., and Mizuno, K. (2002) Mitosis-specific activation of LIM motif-containing protein kinase and roles of cofilin phosphorylation and dephosphorylation in mitosis. *J. Biol. Chem.* **277,** 22093–22102.
106. Yang, E.J., Yoon, J.H., Min, D.S., and Chung, K.C. (2004) LIM kinase 1 activates cAMP-responsive element-binding protein during the neuronal differentiation of immortalized hippocampal progenitor cells. *J. Biol. Chem.* **297**, 8903–8910.
107. Sacchetti, P., Carpentier, R., Segard, P., Olive-Cren, C., and Lefebvre, P. (2006) Multiple signaling pathways regulate the transcriptional activity of the orphan nuclear receptor NURR1. *Nucleic Acids Res.* **34,** 5515–5527.
108. Lee-Hoeflich, S.T., Causing, C.G., Podkowa, M., Zhao, X., Wrana, J.L., and Attisano, L. (2004) Activation of LIMK1 by binding to the BMP receptor, BMPRII, regulates BMP-dependent dendritogenesis. *EMBO J.* **23,** 4792–4801.
109. Aizawa, H., Wakatsuki, S., Ishii, A., Moriyama, K., Sasaki, Y., Ohashi, K., Sekine-Aizawa, Y., Sehara-Fujisawa, A., Mizuno, K., Goshima, Y., and Yahara, I. (2001) Phosphorylation of cofilin by LIM-kinase is necessary for semaphorin 3A growth cone collapse. *Nat. Neurosci.* **4,** 367–373.
110. Heredia, L., Helguera, P., De Olmos, S., Kedikian, G., Sola Vigo, F., LaFerla, F., Staufenbiel, M., De Olmos, J., Busciglio, J., Caceres, A., and Lorzenzo, A. (2006) Phosphorylation of actin-depolymerizing factor/cofilin by LIM-kinase mediates amyloid beta-induced degeneration: a potential mechanism of neuronal dystrophy in Alzheimer's disease. *J. Neurosci.* **26,** 6533–6542.
111. Kruger, R.P., Aurandt, J., and Guan, K.-L. (2005) Semaphorins command cells to move. *Nat. Rev. Mol. Cell Biol.* **6,** 789–800.
112. Tomiyoshi, G., Horita, Y., Nishita, M., Ohashi, K., and Mizuno, K. (2004) Caspase-mediated cleavage and activation of LIM-kinase 1 and its role in apoptotic membrane blebbing. *Genes Cells* **9,** 591–600.
113. Tursun, B., Schluter, A., Peters, M.A., Viehweger, B., Ostendorff, H.P., Soosairajah, J., Drung, A., Bossenz, M., Johnsen, S.A., Schweizer, M., Bernard, O., and Bach, I. (2005) The ubiquitin ligase Rnf6 regulates local LIM kinase 1 levels in axonal growth cones. *Genes Dev.* **19,** 2307–2319.
114. Yokoo, T., Toyoshima, H., Miura, M., Wang, Y., Iida, K.T., Suzuki, H., Sone, H., Shimano, H., Gotoda, T., Nishimori, S., Tanaka, K., and Yamada, N. (2003) p57Kip2 regulates actin dynamics by binding and translocating LIM-kinase 1 to the nucleus. *J. Biol. Chem.* **278,** 52919–52923.
115. Tassabehji, M., Metcalfe, K., Fergusson, W.D., Carette, M.J.A., Dore, J.K., Donnai, D., Read, A.P., Proschel, C., Gutowski, N.J., Mao, X., and Sheer, D. (1996) LIM-kinase deleted in Williams syndrome. *Nat. Genet.* **13,** 272–273.
116. Meng, Y., Zhang, Y., Tregoubov, V., Janus, C., Cruz, L., Jackson, M., Lu, W.-Y., MacDonald, J.F., Wang, J.Y., Falls, D.L., and Jia, Z. (2002) Abnormal spine morphology and enhanced LTP in LIMK-1 knockout mice. *Neuron* **35,** 121–133.
117. Takahashi, H., Koshimizu, U., Miyazaki, J., and Nakamura, T. (2002) Impaired spermatogenic ability of testicular germ cells in mice deficient in the LIM-kinase 2 gene. *Dev. Biol.* **241,** 259.
118. Meng, Y., Takahashi, H., Meng, J., Zhang, Y., Lu, G., Asrar, S., Nakamura, T., and Jia, Z. (2004) Regulation of ADF/cofilin phosphorylation and synaptic function by LIM-kinase. *Neuropharmacology* **47,** 746–754.
119. Ang, L.-H., Chen, W., Yao, Y., Ozawa, R., Tao, E., Yonekura, J., Uemura, T., Keshishian, H., and Hing, H. (2006)

Lim kinase regulates the development of olfactory and neuromuscular synapses. *Dev. Biol.* **293,** 178–190.
120. Schratt, G.M., Tuebing, F., Nigh, E.A., Kane, C.G., Sabatini, M.E., Kiebler, M., and Greenberg, M.E. (2006) A brain-specific microRNA regulates dendritic spine development. *Nature* **439,** 283–289.
121. Aizawa, H., Wakatsuki, S., Ishii, A., Moriyama, K., Sasaki, Y., Ohashi, K., Sekine-Aizawa, Y., Sehara-Fujisawa, A., Mizuno, K., Goshima, Y., and Yahara, I. (2001) Phosphorylation of cofilin by LIM-kinase is necessary for semaphorin 3A-induced growth cone collapse. *Nat. Neurosci*. **4,** 367–373.
122. Jin, Z. and Strittmatter, S.M. (1997) Rac1 mediates collapsin-1-induced growth cone collapse. *J. Neurosci*. **17,** 6256–6263.
123. Jurney, W.M., Gallo, G., Letourneau, P.C., and McLoon, S.C. (2002) Rac1-mediated endocytosis during ephrin-A2- and semaphorin 3A-induced growth cone collapse. *J. Neurosci*. **22,** 6019–6028.
124. Wanigasekara, Y. and Keast, J.R. (2006) Nerve growth factor, glial cell line-derived neurotrophic factor and neurturin prevent semaphorin 3A-mediated growth cone collapse in adult sensory neurons. *Neuroscience* **142,** 369–379.
125. Endo, M., Ohashi, K., and Mizuno, K. (2007) LIM kinase and slingshot are critical for neurite extension. *J. Biol. Chem*. **282,** 13692–13702.
126. Endo, M., Ohashi, K., Sasaki, Y., Goshima, Y., Niwa, R., Uemura, T., and Mizuno, K. (2003) Control of growth cone motility and morphology by LIM kinase and Slingshot via phosphorylation and dephosphorylation of cofilin. *J. Neurosci*. **23,** 2527–2537.
127. Lee-Hoeflich, S., Causing, C.G., Podkowa, M., Zhao, X., Wrana, J.L., and Attisano, L. (2004) Activation of LIMK1 by binding to the BMP receptor, BMPRII, regulates BMP-dependent dendritogenesis. *EMBO J*. **23,** 4792–4801.
128. Wen, Z., Han, L., Bamburg, J.R., Shim, S., Ming, G.L., and Zheng, J.Q. (2007) BMP gradients steer nerve growth cones by a balancing act of LIM kinase and Slingshot phosphatase on ADF/cofilin. *J. Cell Biol*. **178,** 107–119.
129. Semenova, E., Wang, X., Jablonski, M.M., Levorse, J., and Tilghman, S.M. (2003) An engineered 800 kilobase deletion of Uchl3 and Lmo7 on mouse chromosome 14 causes defects in viability, postnatal growth and degeneration of muscle and retina. *Hum. Mol. Genet*. **12,** 1301–1312.
130. Holaska, J.M., Rais-Bahrami, S., and Wilson, K.L. (2006) Lmo7 is an emerin-binding protein that regulates the transcription of emerin and many other muscle-relevant genes. *Hum. Mol. Genet.* **15,** 3459–3472.
131. Rozenblum, E., Vahteristo, P., Sandberg, T., Bergthorsson, J., Syrjakoski, K., Weaver, D., Haraldsson, K., Johannsdottir, H., Vehmanen, P., Nigam, S., Golberger, N., Robbins, C., Pak, E., Dutra, A., Gillander, E., Stephan, D., Bailey-Wilson, J., Juo, S.-H., Kainu, T., Arason, A., Barkardottir, R., Nevanlinna, H., Borg, A., and Kallioniemi, O.P. (2002) A genomic map of a 6-Mb region at 13q21-q22 implicated in cancer development: identification and characterization of candidate genes. *Hum. Genet*. **110,** 111–121.
132. Putilina, T., Jaworski, C., Gentleman, S., McDonald, B., Kadiri, M., and Wong, P. (1998) Analysis of a human cDNA containing a tissue-specific alternatively spliced LIM domain. *Biochem. Biophys. Res. Commun*. **252,** 433–439.
133. Nakamura, H., Mukai, M., Komatsu, K., Tanaka-Okamoto, M., Itoh, Y., Ishizaki, H., Tatsuta, M., Inoue, M., and Miyoshi, J. (2005) Transforming growth factor-beta1 induces LMO7 while enhancing the invasiveness of rat ascites hepatoma cells. *Cancer Lett.* **220,** 95–99.
134. Jin, J., Cardozo, T., Lovering, R.C., Elledge, S.J., Pagano, M., and Harper, J.W. (2004) Systematic analysis and nomenclature of mammalian F-box proteins. *Genes Dev*. **18,** 2573–2580.
135. Kurihara, L.J., Semenova, E., Miller, W., Ingram, R.S., Guan, X.-J., and Tilghman, S.M. (2002) Candidate genes required for embryonic development: a comparative analysis of distal mouse chromosome 14 and human chromosome 13q22. *Genomics* **79,** 154–161.
136. Kang, S., Xu, H., Duan, X., Liu, J.-J., He, Z., Yu, F., Zhou, S., Meng, X.-Q., Cao, M., and Kennedy, G.C. (2000) PCD1, a novel gene containing PDZ and LIM domains, is overexpressed in several human cancers. *Cancer Res*. **60,** 5296–5302.
137. Furuya, M., Tsuji, N., Endoh, T., Moriai, R., Kobayashi, D., Yagihashi, A., and Watanabe, N. (2002) A novel gene containing PDZ and LIM domains, PCD1, is overexpressed in human colorectal cancer. *Anticancer Res*. **22,** 4183–4186.
138. Sasaki, M., Tsuji, N., Furuya, M., Kondoh, K., Kamagata, C., Kobayashi, D., Yagihashi, A., Watanabe, N. (2003) PCD1, a novel gene containing PDZ and LIM domains, is overexpressed in human breast cancer and linked to lymph node metastasis. *Anticancer Res*. **23(3B),** 2717–2721.
139. Lindvall, J.M., Blomberg, K.E.M., Wennborg, A., and Smith, C.I.E. (2005) Differential expression and molecular characterisation of Lmo7, Myo1e, Sash1, and Mcoln2 genes in Btk-defective B-cells. *Cell. Immunol*. **235,** 46–55.
140. Linask, K.K. and VanAuker, M. (2007) A role for the cytoskeleton in heart looping. *TheScientificWorldJOURNAL* **7,** 280–298.
141. Vardouli, L., Moustakas, A., and Stournaras, C. (2005) LIM-kinase 2 and cofilin phosphorylation mediate actin cytoskeleton reorganization induced by transforming growth factor-{beta}. *J. Biol. Chem*. **280,** 11448–11457.
142. Litingtung, Y. and Chiang, C. (2000) Control of Shh activity and signaling in the neural tube. *Dev. Dyn*. **219,** 143–154.
143. Bozulic, L.D., Malik, M.T., Powell, D.W., Nanez, A., Link, A.J., Ramos, K.S., and Dean, W.L. (2007) Plasma membrane Ca2+-ATPase associates with CLP36, α-actinin and actin in human platelets. *Thromb. Haemost*. **97,** 587–597.
144. Barres, R., Gonzalez, T., Marchand-Brustel, Y.L., and Tanti, J.-F. (2005) The interaction between the adaptor protein

APS and Enigma is involved in actin organisation. *Exp. Cell Res*. **308,** 334–344.
145. Takahashi, T., Aoki, S., Nakamura, T., Koshimizu, U., Matsumoto, K., and Nakamura, T. (1997) Xenopus LIM motif-containing protein kinase, Xlimk1, is expressed in the developing head structure of the embryo. *Dev. Dyn*. **209,** 196–205.
146. Hiraoka, J., Okano, I., Higuchi, O., Yang, N., and Mizuno, K. (1996) Self-association of LIM-kinase 1 mediated by the interaction between an N-terminal LIM domain and a C-terminal kinase domain. *FEBS Lett*. **399,** 117–121.
147. Yang, N. and Mizuno, K. (1999) Nuclear export of LIM-kinase 1, mediated by two leucine-rich nuclear-export signals within the PDZ domain. *Biochem. J.* **338,** 793–798.
148. Rosso, S., Bollati, F., Bisbal, M., Peretti, D., Sumi, T., Nakamura, T., Quiroga, S., Ferreira, A., and Caceres, A. (2004) LIMK1 regulates Golgi dynamics, traffic of Golgi-derived vesicles, and process extension in primary cultured neurons. *Mol. Biol. Cell* **15,** 3433–3449.
149. Kobayashi, M., Nishita, M., Mishima, T., Ohashi, K., and Mizuno, K. (2006) MAPKAPK-2-mediated LIM-kinase activation is critical for VEGF-induced actin remodeling and cell migration. *EMBO J*. **25,** 713–726.
150. Acevedo, K., Moussi, N., Li, R., Soo, P., and Bernard, O. (2006) LIM kinase 2 is widely expressed in all tissues. *J. Histochem. Cytochem*. **54,** 487–501.
151. Goyal, P., Pandey, D., Behring, A., and Siess, W. (2005) Inhibition of nuclear import of LIMK2 in endothelial cells by protein kinase C-dependent phosphorylation at Ser-283. *J. Biol. Chem.* **280,** 27569–27577.
152. Cuppen, E., Gerrits, H., Pepers, B., Wieringa, B., and Hendriks, W. (1998) PDZ motifs in PTP-BL and RIL bind to internal protein segments in the LIM domain protein RIL. *Mol. Biol. Cell* **9,** 671–683.
153. Zou, P., Pinotsis, N., Lange, S., Song, Y.-H., Popov, A., Mavridis, I., Mayans, O.M., Gautel, M., and Wilmanns, M. (2006) Palindromic assembly of the giant muscle protein titin in the sarcomeric Z-disk. *Nature* **439,** 229–233.
